# Numerical study of magnetohydrodynamic nanofluid flow over a porous surface under convective heating and zero mass flux conditions

**DOI:** 10.1186/s11671-026-04788-z

**Published:** 2026-07-20

**Authors:** Showkat Ahmad Lone, Chetan Swarup, Laila A. AL-Essa, Zehba Raizah, Anwar Saeed, Gabriella Bognár

**Affiliations:** 1 Department of Basic Sciences, College of Science and Theoretical Studies, Saudi Electronic University, (Jeddah-M), Riyadh, 11673 Saudi Arabia; 2 Department of Mathematical Sciences, College of Science, Princess Nourah bint Abdulrahman University, P.O. Box 84428, Riyadh, 11671 Saudi Arabia; 3 Department of Mathematics, Faculty of Science, King Khalid University, Abha, Saudi Arabia; 4 Department of Computer Engineering, Biruni University, Istanbul, 34010 Turkey; 5 Institute of Machine and Product Design, University of Miskolc, Miskolc-Egyetemváros, 3515 Hungary

**Keywords:** Nanofluid, Porous media, MHD, Thermophoresis, Brownian motion, Activation energy, Thermal convective, Zero mass flux conditions

## Abstract

This work examines magnetohydrodynamic (MHD) flow of a nanoliquid through on a porous surface. The mathematical model includes the collective effects of thermophoresis and Brownian motion to accurately describe nanoparticle behavior. It further analyzes the effect of Arrhenius activation energy on chemically reactive species. The flow is administrated by a convective heating condition, while the concentration field is subjected to a physically realistic zero mass flux condition. The bvp4c approach is used in this work to solve the modeled equations in dimension-free form. It is revealed as outcomes of this work that, for augmentation in magnetic factor, inter-particle spacing and porosity factor there is lessening in primary and secondary flows. Both the velocities augmented with progression in radius of nanoparticles. Thermal profiles augmented with growth in thermal Biot number, and magnetic factor while declined with augmentation in thermal relaxation time factor. Concentration panels augmented with progression in thermophoresis factor and activation energy factor while weakened with augmentation in Schmidt number and Brownian motion factor. A comparative analysis with established results confirms the accuracy and validity of the present model. The close agreement between our numerical outputs and the published data verifies the correctness of the solution methodology and the physical consistency of the formulated problem. This study demonstrates that the simultaneous adjustment of inter-particle spacing and nanoparticle size provides a strategic approach for enhancing thermal performance relative to pumping in nanofluid-based systems, with immediate ramifications for the design of advanced microelectronics coolants and magnetically guided delivery platforms.

## Introduction

Nanofluid flow, a specialized domain within fluid dynamics and nanotechnology, involves the study of suspensions containing nanoparticles dispersed within a conventional base fluid. The defining characteristic of these flows is the intense alteration of the base fluid’s thermophysical properties as discovered first by Choi [1]. Sometimes even minimal nanoparticle volumetric concentrations (often below 5%) can significantly enhance thermal conductivities and heat transfer coefficients. Mondal and Pal [2] studied magneto nanofluid flow on an inclined expanding surface. Nazari et al. [3] studied thermally radiative and convective nanofluid flow on an inclined contracting/extending surface with suction of mass. Mondal and Pal [4] studied the impression of activation energy and varying thermal conductance on magnetized nanofluid flow with effects of microorganisms. The dynamics of nanofluid flow are used in coolant of electronic devices, nuclear reactor and coolant of auto engine. Ragavi et al. [5] discussed transition flow for nanofluid on a radially elongating surface with Ohmic and dissipative heating effects. Khan et al. [6] discussed the hydrothermal performance for nanofluid flow on an elongating surface using dissipative effects. Research in this field is intensely application-driven, primarily targeting next-generation cooling solutions for high-heat-flux environments such as microelectronics, advanced nuclear reactors, and concentrated solar thermal systems. However, practical implementation faces substantial challenges, including achieving stable, homogeneous suspensions that resist aggregation and sedimentation over time. Nanofluids are used to achieve unprecedented thermal management performance, thereby bridging the gap between nanoscale science and macroscopic systems. Yaseen et al. [7] examined numerically the nanofluid flow with irregular sink/source effects. Ahmad et al. [8] scrutinized MHD flow using a penta-hybrid nanofluid. Their research focused on the enhanced thermal characteristics and complex dynamics resulting from the suspension of five distinct nanoparticle types within the base fluid.

Fluid flow through porous media, a foundational concept in fields ranging from petroleum engineering to groundwater hydrology and biomedical engineering, describes the movement of liquids within a complex, solid matrix containing interconnected void spaces. This matrix, or porous space, is characterized by key properties like porosity, which defines the fraction of void volume, and permeability, an intrinsic measure of the medium’s ability to transmit fluid under an applied pressure gradient. Pal et al. [9] examined mass and thermal transferences for thin film nanofluid flow on time-dependent expanding sheet immersed by porous medium and chemical reactivity. Luo et al. [10] considered particles hydrodynamics fluid flow on a penetrable sheet using ocean and costal engineering applications. Shah et al. [11] scrutinized the flow of fluid on a permeable surface. Their investigation specifically considered the collective influence of fluid flow slip at the boundary and thermal radiative heat transfer on the fluid’s hydrodynamic and thermal boundary layer characteristics. The governing principles for such flows are often described by Darcy’s Law. The applications of this science are vast and critical: it enables the extraction of oil and gas from reservoir rocks, the management of aquifer recharge and contaminant transport, the design of industrial filters and catalytic reactors, and even the understanding of physiological processes like blood perfusion in tissues or fluid movement in bones [12, 13]. Sreenivasulu et al. [14] discussed the thermal features of fluid flow on a cone with effects of heat source on flow system. Ashfaq et al. [15] considered magnetized flow on a permeable surface. Their analysis focused on effects of motile microorganisms and specific thermal boundary conditions, examining the complex interplay between magnetic forces, bio-convection, and thermal constraints in the fluid system. Ahmadi Azar et al. [16] scrutinized comprehensively Casson magnetohydrodynamic fluid flow on a rectangular permeable sheet in a contacting and expanding channel.

Magneto-hydrodynamics (MHD) examines the flow of electrically conductive fluids, with magnetic effects. When a conductive fluid flows through a magnetic field, it generates a braking force (the Lorentz force) that pushes against the flow and changes how it moves. This fundamental interaction introduces reflective complexities, transforming simple laminar flows into scenarios rich with anisotropic viscosity, where the fluid’s resistance to motion depends on direction relative to the magnetic field lines. Poornima et al. [17] discoursed the production of entropy for radiative magnetized nanofluid flow through a permeable surface on a movable wedge. Lone et al. [18] discoursed thermal examination of fluid flow on a varying permeable space. The practical ramifications of these principles are vast, enabling technologies from controlled nuclear fusion, where magnetic fields confine superheated plasma, to industrial processes like electromagnetic casting of metals, which allows for contactless, precise shaping. Sreenivasulu et al. [19] scrutinized computationally the magnetized nanofluid flow on an elongating surface. The mass and thermal transport characteristics of an unsteady magnetized nanofluid flowing along a surface were studied by Ragavi et al. [20]. The flow of a magnetized fluid on a moving vertical permeable medium, including the effects of viscous dissipation, was discussed by Lakshmi et al. [21]. Ganji et al. [22] considered the time-dependent MHD fluid flow of a penta-hybrid nanofluid. The Lorentz force works as resistive brake, directly opposing fluid motion. This expressively reduces the velocity profile, flattening it in the core of the flow while creating steep gradients in thin momentum layers as discussed by Zeeshan et al. [23]. The governing equations reveal that the strength of this magneto-fluidic coupling is primarily dictated by two key dimensionless parameters: the Hartmann number and the magnetic Reynolds number. For temperature, the damping of fluid motion and turbulence suppresses convective mixing, leading to reduced heat transfer rates. Vinothkumar et al. [24] examined thermal transportation and flow dynamics for magnetized nanofluid on a cone with slip impression.

The interaction of thermophoresis and Brownian motion fundamentally governs the complex behavior of nanoparticle suspensions in fluid flows, particularly in advanced thermal systems utilizing nanofluids. Thermophoresis, describes the phenomenon where suspended particles experience a force and consequently migrate along a temperature gradient, typically from a hotter region to a cooler one, driven by imbalances in molecular kinetic energy on opposing particle surfaces. Conversely, Brownian motion describes the perpetual, random walk of nanoparticles due to continuous bombardment by the molecules of the base fluid, a kinetic process that enhances particle dispersion and promotes mixing. These effects [25–28] are frequently used in various fluid flows specifically when nanoparticles are suspended in base fluid. Taha et al. [29] studied magnetohydrodynamics fluid flow on a wedge with Brownian motion and thermophoresis effects. In a flowing nanofluid, these two mechanisms are fundamentally coupled and compete: thermophoresis tends to generate particle concentration gradients by driving particles toward cooler zones, while Brownian diffusion works to homogenize the particle distribution as examined by Rasool et al. [30]. This dynamic is mathematically captured in modified conservation equations, where the particle flux includes separate terms for thermophoretic and Brownian diffusion coefficients, the latter being strongly temperature-dependent. The significance of this coupling extends beyond mere particle redistribution; it critically modifies the thermal and hydrodynamic boundary layers. For instance, in a boundary layer flow on a heated surface, thermophoresis can deplete nanoparticles from the hot wall, forming a concentrated particle layer farther away, while Brownian motion constantly works to counter this stratification. Swami et al. [31] studied thermal transfer for fluid flow on a wedge. Varshegaa et al. [32] examined mass and thermal transferences for magnetized fluid flow using thermophoresis and Brownian motion mechanisms.

The incorporation of activation energy into models of fluid flow introduces a vital dimension of chemically reactive processes, fundamentally transforming the analysis of systems. Shaheen et al. [33] discussed machine learning technique for fluid flow with activation energy. In fluid dynamics, this is particularly relevant for reactive flows involving combustion, polymerization, catalytic surface reactions, or biological processes like enzymatic degradation in bioreactors [34–36]. Khouqeer et al. [37] considered fluid flow on a gyrating disk with activation energy. When a fluid containing reactants flows, the local temperature and species concentration fields shaped by advection, diffusion, and conduction directly dictate the local reaction rate, which is exponentially sensitive to temperature via the Arrhenius factor as revealed by Wantao et al. [38]. This creates a powerful, nonlinear feedback loop: exothermic reactions release heat, elevating local temperature, which exponentially accelerates further reaction, potentially leading to ignition, thermal runaway, or distinct flame fronts. Conversely, endothermic reactions can suppress temperature rises and stabilize flows. Applications are intensely important, spanning the design of efficient, low-emission combustors in jet engines and gas turbines, the optimization of chemical vapor deposition reactors for material synthesis, the management of underground combustion for heavy oil recovery, and the understanding of metabolic processes in tissue engineering. The presence of activation energy thus elevates fluid flow from a purely physical phenomenon to a dynamic, thermo-chemical system where fluid mechanics, thermodynamics, and chemical kinetics are inseparably intertwined as Thumma et al. [39]. Zhang et al. [40] used activation energy thermally radiative effects subject to bio-convection flow of nanoparticles on a shrinking and expanding disk.

This analysis demonstrates a comprehensive investigation of MHD bio-convective flow of a gold-water (Au-H_2_O) nanofluid past a permeable extending sheet. With unique contributions in the following areas, this paper advances the subject through a comprehensive integration of physics phenomena suitable for biomedical and energy applications:

- This work is novel in its strategic synthesis of the Cattaneo–Christov flux theory, activation energy and gyrotactic bioconvection within homogeneous model of Au-water nanofluid subject to MHD, and thermal convective heating. This integration is critical for real-world problems like magnetic-hyperthermia treatment with drug-carrying microorganisms.
- A theoretical improvement in the enforcement of realistic zero-mass flux condition for the Au nanoparticle at the boundary. This guarantees that the balance of Brownian motion and thermophoresis is the only factor controlling the nanoparticle volume fraction, making the model more applicable to systems such as surface-based nanofluid cooling systems.
- This model incorporates a viscosity correlation that depends on size and volume fraction of Au nanoparticle, crucial for accuracy, dispersed in water base fluid.

**Research questions:**

**Q1:** How does the implementation of Cattaneo–Christov flux theory and gyrotactic bioconvection influence the thermal, molar concentration and microorganism transport in a water-based Au nanofluid flow?

**Q2:** What is the impact of physically realistic zero-mass flux condition, governed solely by the thermophoresis and Brownian motion, on mass and thermal transference characteristics of the water-based Au nanofluid flow?

**Q3:** How do variations in inter-particle spacing and nanoparticle radius influence the skin friction and velocity profiles of a water-based Au nanofluid flow?

To establish this novel framework, the mathematical formulation is detailed in Sect. 2. The numerical methodology for solving the resultant equations is designated in Sect. 3, with its validation presented in Sect. 4. A comprehensive discussion of the results follows in Sect. 5, and the principal conclusions are summarized in Sect. 6.

## Problem formulation

**Fig. 1:**
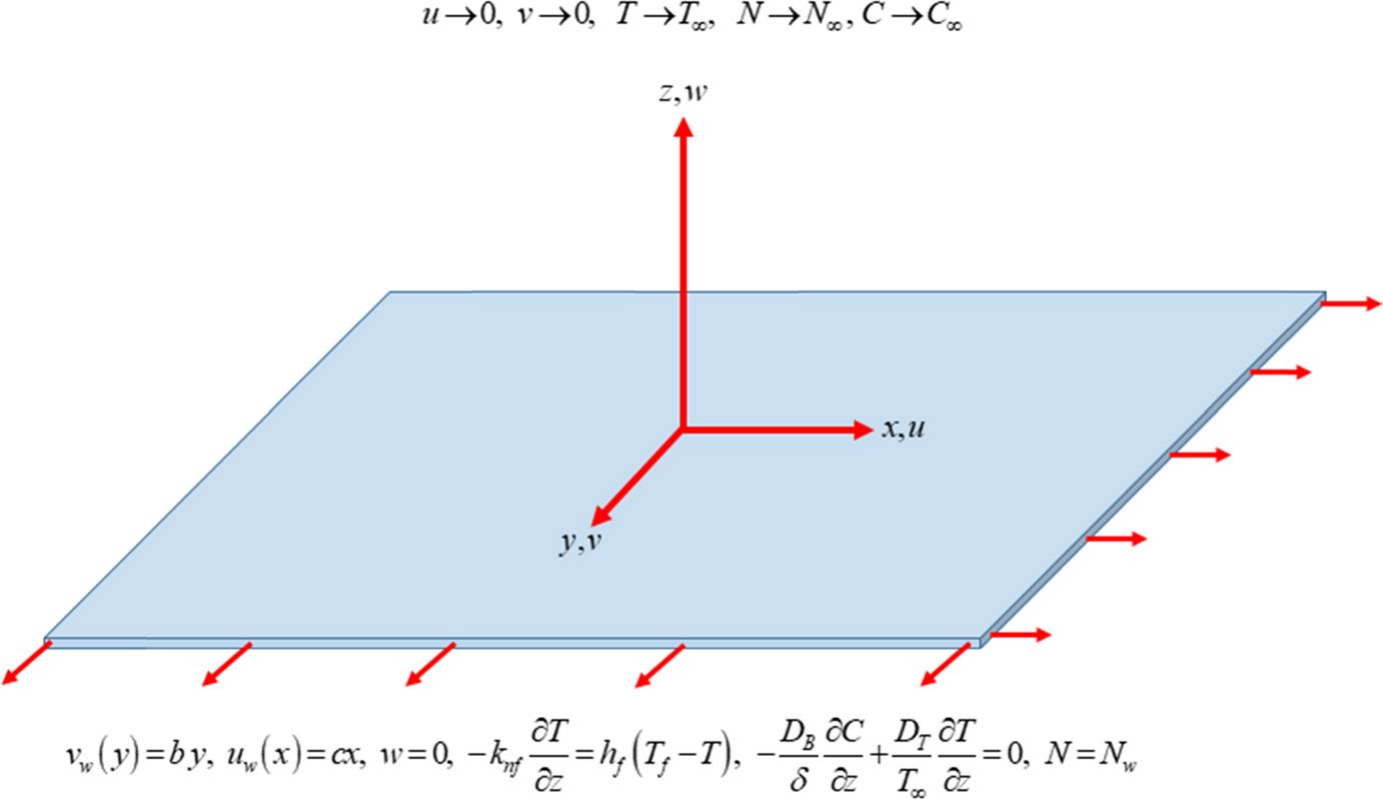
Schematic diagram of the flow problem

Consider nanofluid flow on permeable surface which is expanding in both x- and y-directions simultaneously. Following assumption are made in this study:

- The expanding velocities of sheet along coordinate axes are $u_{w} \left( x \right) = cx$ and $v_{w} \left( y \right) = by$ with $c\left( { > 0} \right)$ and $b\left( { > 0} \right)$ as fixed numbers.
- A magnetic force of intensity $B\left( {0,\,\,0,\,\,B_{0} } \right)$ is applied in normal direction to flow system as examined in Fig. 1.
- Both the surface and free stream values for temperatures, concentrations, and microorganism concentrations are expressed symbolically as $T_{w} ,\,\,T_{\infty }$, $C_{w} ,\,\,C_{\infty }$ and $N_{w} ,\,\,N_{\infty }$.
- Additionally, a zero-mass-flux condition is employed to examine heat and mass transfer rates.
- This study examines the flow of a bioconvective nanofluid, incorporating the Cattaneo–Christov model and the influence of chemical reactivity.

From the stated suppositions we have [41, 42]

1 $$ \frac{\partial \,\,u}{{\partial \,x}} + \frac{\partial \,\,v}{{\partial \,y}} + \frac{\partial \,\,w}{{\partial \,z}} = 0, $$

2 $$ u\frac{\partial \,\,u}{{\partial \,x}} + v\,\frac{\partial \,u}{{\partial \,\,y}} + w\frac{\partial \,\,u}{{\partial \,z}} = \frac{{\mu_{nf} }}{{\rho_{nf} }}\frac{{\partial^{2} u}}{{\partial z^{2} }} - \frac{1}{{K^{*} }}\frac{{\mu_{nf} }}{{\rho_{nf} }}u - \frac{{\sigma_{nf} }}{{\rho_{nf} }}B_{0}^{2} u,\, $$

3 $$ u\frac{\partial \,\,v}{{\partial \,x}} + v\,\frac{\partial \,\,v}{{\partial \,y}} + w\frac{\partial \,\,v}{{\partial \,\,z}} = \frac{{\mu_{nf} }}{{\rho_{nf} }}\frac{{\partial^{2} v}}{{\partial z^{2} }} - \frac{1}{{K^{*} }}\frac{{\mu_{nf} }}{{\rho_{nf} }}v - \frac{{\sigma_{nf} }}{{\rho_{nf} }}B_{0}^{2} v, $$

4 $$ \begin{gathered} u\frac{{\partial T}}{{\partial x}} + v\frac{{\partial T}}{{\partial y}} + w\frac{{\partial T}}{{\partial z}} + \lambda _{T} \left\{ {u\frac{{\partial u}}{{\partial x}}\frac{{\partial T}}{{\partial x}} + v\frac{{\partial u}}{{\partial y}}\frac{{\partial T}}{{\partial x}} + w\frac{{\partial w}}{{\partial z}}\frac{{\partial T}}{{\partial x}} + u\frac{{\partial v}}{{\partial x}}\frac{{\partial T}}{{\partial y}} + v\frac{{\partial v}}{{\partial y}}\frac{{\partial T}}{{\partial y}} + w\frac{{\partial v}}{{\partial z}}\frac{{\partial T}}{{\partial y}}} \right. \hfill \\ + u\frac{{\partial w}}{{\partial x}}\frac{{\partial T}}{{\partial z}} + v\frac{{\partial T}}{{\partial z}}\frac{{\partial w}}{{\partial {\mkern 1mu} y}} + w\frac{{\partial T}}{{\partial {\mkern 1mu} z}}\frac{{\partial w}}{{\partial {\mkern 1mu} z}} + 2vu\frac{{\partial ^{2} {\mkern 1mu} T}}{{\partial {\mkern 1mu} x{\mkern 1mu} \partial {\mkern 1mu} y}} + 2wv\frac{{\partial ^{2} T}}{{\partial {\mkern 1mu} y{\mkern 1mu} \partial {\mkern 1mu} z}} \hfill \\ + 2wu\frac{{\partial ^{2} T}}{{\partial {\mkern 1mu} x{\mkern 1mu} \partial {\mkern 1mu} z}} + u^{2} \frac{{\partial ^{2} T}}{{\partial {\mkern 1mu} x^{2} }} + v^{2} \frac{{\partial ^{2} T}}{{\partial {\mkern 1mu} y^{2} }} + \left. {w^{2} \frac{{\partial ^{2} T}}{{\partial {\mkern 1mu} z^{2} }}} \right\} = \frac{{k_{{nf}} }}{{\left( {\rho C_{p} } \right)_{{nf}} }}\frac{{\partial ^{2} T}}{{\partial z^{2} }} \hfill \\ + \frac{{\left( {\rho C_{p} } \right)_{{np}} }}{{\left( {\rho C_{p} } \right)_{{nf}} }}\left( {\frac{{D_{{{\kern 1pt} B}} }}{\delta }\frac{{\partial {\mkern 1mu} C}}{{\partial {\mkern 1mu} z}}\frac{{\partial {\mkern 1mu} T}}{{\partial {\mkern 1mu} z}} + \frac{{D{\mkern 1mu} _{T} }}{{T_{{{\kern 1pt} \infty }} }}\left( {\frac{{\partial {\mkern 1mu} T}}{{\partial {\mkern 1mu} z}}} \right)^{2} } \right), \hfill \\ \end{gathered} $$

5 $$ \begin{aligned} & u\frac{\partial \,C}{{\partial \,x}} + v\frac{\partial \,C}{{\partial \,y}} + w\frac{\partial \,C}{{\partial \,z}} + \lambda_{C} \left\{ {u\frac{\partial \,C}{{\partial x}}\frac{\partial u}{{\partial x}} + v\frac{\partial C}{{\partial x}}\frac{\partial \,u}{{\partial y}} + w\frac{\partial C}{{\partial x}}\frac{\partial w}{{\partial z}} + u\frac{\partial C}{{\partial y}}\frac{\partial v}{{\partial x}} + v\frac{\partial C}{{\partial y}}\frac{\partial v}{{\partial y}} + w\frac{\partial C}{{\partial y}}\frac{\partial v}{{\partial z}}} \right. + \\ & \quad u\frac{\partial C}{{\partial z}}\frac{\partial w}{{\partial x}} + v\frac{\partial C}{{\partial z}}\frac{\partial w}{{\partial y}} + w\frac{\partial C}{{\partial z}}\frac{\partial w}{{\partial z}} + 2u\,v\frac{{\partial^{2} C}}{\partial x\partial y} + 2v\,w\frac{{\partial^{2} C}}{\partial y\partial z} + 2u\,w\frac{{\partial^{2} C}}{\partial x\partial z} + u^{2} \frac{{\partial^{2} C}}{{\partial x^{2} }} + v^{2} \frac{{\partial^{2} C}}{{\partial y^{2} }} + \left. {w^{2} \frac{{\partial^{2} C}}{{\partial z^{2} }}} \right\} \\ & \quad \quad = D_{B} \frac{{\partial^{2} \,C}}{{\partial \,z^{2} }} + \frac{{\partial^{2} T}}{{\partial z^{2} }}\frac{{\delta D_{T} }}{{T_{\infty } }} - K_{1} \left( {C - C_{\infty } } \right)\left( {\frac{T}{{T_{\infty } }}} \right)^{n} \exp \left( {\frac{{ - E_{a} }}{kT}} \right), \\ \end{aligned} $$

6 $$ u\,\frac{\partial \,N}{{\partial \,x}} + v\,\,\frac{\partial \,N}{{\partial \,y}} + w\frac{\partial \,N}{{\partial \,z}} + \frac{{b\,W_{c} }}{{C_{\infty } }}\left[ {\frac{\partial }{\partial \,z}\left( {N\frac{\partial \,C}{{\partial \,z}}} \right)} \right] = D_{m} \frac{{\partial^{2} N}}{{\partial z^{2} }}, $$

The pertinent conditions at the boundaries are given by:

7 $$ \begin{aligned} & \left. \begin{gathered} u_{w} \,\left( {\,x\,} \right) = x\,c\,,\,\,\, \hfill \\ v_{w} \left( y \right) = y\,b\,,\,\,\,\, \hfill \\ w = 0,\,\,\, \hfill \\ - k_{nf} \frac{\partial \,T}{{\partial \,z}} = h\,_{f} \left( {T\,_{f} - T} \right),\,\,\, \hfill \\ - \frac{{D_{B} }}{\delta }\frac{\partial C}{{\partial z}} + \frac{{D_{T} }}{{T_{\infty } }}\frac{\partial T}{{\partial z}} = 0, \hfill \\ N = N_{w} \hfill \\ \end{gathered} \right\}\,\,\,\,\,\,\,\,at\,\,\,z = 0, \\ & \,\,\,\,\,\,\,\,\,\,\,\,\,\,\,\,\,\,\,\,\,\,\,\,\,\,\,\,\,\,\,\,\,\,\,\,\,\left. \begin{gathered} \,\,u \to 0,\,\,\,v \to 0,\,\,\,\, \hfill \\ \,\,\,\,\,\,\,\,\,\,T \to T_{\infty } ,\,\,\,\, \hfill \\ \,\,\,\,\,\,\,\,\,N \to N_{\infty } \,,\,\, \hfill \\ \,\,\,\,\,\,\,\,\,\,C \to C_{\infty } ,\, \hfill \\ \end{gathered} \right\}\,\,\,\,\,as\,\,\,\,z \to \infty . \\ \end{aligned} $$

Above, flow components are $u,v,w$, $\mu$ is dynamic viscosity, $\rho$ is the density, $K^{*}$ is porosity coefficient, $\sigma$ is electrical conductivity, $\lambda_{C}$ is dimensional mass relaxation time factor, $B_{0}$ is magnetic field, $E_{a}$ is the activation energy coefficient, $\lambda_{T}$ is dimensional thermal relaxation time factor, $C_{p}$ is specific heat, $n$ is power index, $W_{c}$ is maximum swimming speed of cell, $K_{1}$ is chemical reaction coefficient, $D_{m}$ is microorganism diffusion and $h_{f}$ is heat transfer coefficient.

The thermal and physical properties are explained in Eqs. (8) and (9) while their numerical values are illustrated in Table 1.

8 $$ \frac{{\mu_{nf} }}{{\mu_{f} }} = 1 + 2.5\varphi + 4.5\left( {\frac{1}{{\frac{h}{{d_{p} }}\left( {2 + \frac{h}{{d_{p} }}} \right)\left( {1 + \frac{h}{{d_{p} }}} \right)^{2} }}} \right). $$

**Table 1:** Thermo-physical features of water and Au [42]

	$C_{p} \left[ {\mathrm{J/kgK}} \right]$	$\rho \left[ {{\mathrm{kg/m}}^{{3}} } \right]$	$k\left[ {\mathrm{W/mK}} \right]$	$\sigma \left[ {\mathrm{S/m}} \right]$
Water	$4179$	$997.1$	$0.613$	$0.005$
Au	129	$19320$	$314$	$4.100 \times 10^7$

In Eq. (8), $\varphi ( > 0)$ is volume fraction of nanoparticle, $d_{p}$ is the nanoparticle radius, and $h$ is inter-particle spacing.

Further, we have

9 $$ \begin{aligned} \frac{{\rho_{nf} }}{{\rho_{f} }} & = 1 - \varphi + \varphi \frac{{\rho_{np} }}{{\rho_{f} }},\,\,\,\,\frac{{\left( {\rho C_{p} } \right)_{nf} }}{{\left( {\rho C_{p} } \right)_{f} }} = 1 - \varphi + \varphi \frac{{\left( {\rho C_{p} } \right)_{np} }}{{\left( {\rho C_{p} } \right)_{f} }},\,\,\,\frac{{\nu_{nf} }}{{\nu_{f} }} = \frac{{\mu_{nf} /\mu_{f} }}{{\rho_{nf} /\rho_{f} }}, \\ \frac{{k_{nf} }}{{k_{f} }} & = \frac{{k\,_{npf} - 2\,\varphi \left( {k_{f} - k_{np} } \right) + 2\,k}}{{k\,_{np} + \varphi \,\left( {k_{f} - k_{np} } \right) + 2\,k_{f} }},\,\,\,\,\frac{{\sigma_{nf} }}{{\sigma_{f} }} = 1 + \frac{{3\left( {\frac{{\sigma \,_{n\,p} }}{{\sigma_{f} }} - 1} \right)\varphi }}{{\left( {\frac{{\sigma \,_{n\,p} }}{{\sigma_{f} }} + 2} \right) + \varphi \left( {1 - \frac{{\sigma_{np} }}{{\sigma_{f} }}} \right)}}. \\ \end{aligned} $$

The variables transformations are:

10 $$ \begin{aligned} u & = cxf^{\prime}\left( \eta \right),v = cyg^{\prime}\left( \eta \right),\,\,w = - \sqrt {c\nu_{f} } \left\{ {\left( {f\left( \eta \right) + g\left( \eta \right)} \right)} \right\}, \\ \theta \left( \eta \right) & = \frac{{T - T_{\infty } }}{{T_{f} - T_{\infty } }},\,\,\,\,\phi \left( \eta \right) = \frac{{C - C_{\infty } }}{{C_{\infty } }},\,\,\,\,\,\,\,\eta = z\sqrt {\frac{c}{{\nu_{f} }}} \,,\,\,\,\,\psi \left( \eta \right) = \frac{{N - N_{\infty } }}{{N_{w} - N_{\infty } }}. \\ \end{aligned} $$

Using Eq. (10) we have from above as:

11 $$ \frac{{A_{1} }}{{A_{2} }}f^{\prime\prime\prime}\left( \eta \right) + \left( {f\left( \eta \right) + g\left( \eta \right)} \right)f^{\prime\prime}\left( \eta \right) - f^{{\prime}{2}} \left( \eta \right) - \frac{{A_{3} }}{{A_{2} }}Mf^{\prime}\left( \eta \right) - \frac{{A_{1} }}{{A_{2} }}Kf^{\prime}\left( \eta \right) = 0, $$

12 $$ \frac{{A_{1} }}{{A_{2} }}g^{\prime\prime\prime}\left( \eta \right) - g^{{\prime}{2}} \left( \eta \right) + \left( {g\left( \eta \right) + f\left( \eta \right)} \right)g^{\prime\prime}\left( \eta \right) - \frac{{A_{3} }}{{A_{2} }}Mg^{\prime}\left( \eta \right) - \frac{{A_{1} }}{{A_{2} }}Kg^{\prime}\left( \eta \right) = 0, $$

13 $$ \begin{aligned} & \frac{{A_{4} }}{{A_{5} }}\theta^{\prime\prime}\left( \eta \right) + \Pr \left( {f\left( \eta \right) + g\left( \eta \right)} \right)\theta^{\prime}\left( \eta \right) + \frac{\Pr }{{A_{5} }}\left( {Nb\phi^{\prime}\left( \eta \right)\theta^{\prime}\left( \eta \right) + Nt\theta^{{\prime}{2}} \left( \eta \right)} \right) \\ & \quad - \Pr \delta_{T} \left\{ {\left( {g\left( \eta \right) + f\left( \eta \right)} \right)\left( {g^{\prime}\left( \eta \right) + f^{\prime}\left( \eta \right)} \right)\theta^{\prime}\left( \eta \right) + \left( {g\left( \eta \right) + f\left( \eta \right)} \right)^{2} \theta^{\prime\prime}\left( \eta \right)} \right\} = 0, \\ \end{aligned} $$

14 $$ \begin{aligned} & \phi^{\prime\prime}\left( \eta \right) + \frac{Nt}{{Nb}}\theta^{\prime\prime}\left( \eta \right) + Sc\left( {f\left( \eta \right) + g\left( \eta \right)} \right)\theta^{\prime}\left( \eta \right) - K_{r} Sc\phi \left( \eta \right)\left( {1 + \sigma \theta \left( \eta \right)} \right)^{n} e^{{\left( { - \frac{E}{1 + \sigma \theta \left( \eta \right)}} \right)}} \\ & \quad - Sc\delta_{C} \left\{ {\left( {f\left( \eta \right) + g\left( \eta \right)} \right)\left( {f^{\prime}\left( \eta \right) + g^{\prime}\left( \eta \right)} \right)\phi^{\prime}\left( \eta \right) + \left( {g\left( \eta \right) + f\left( \eta \right)} \right)^{2} \phi^{\prime\prime}\left( \eta \right)} \right\} = 0, \\ \end{aligned} $$

15 $$ \psi^{\prime\prime}\left( \eta \right) + Lb\left( {f\left( \eta \right) + g\left( \eta \right)} \right)\psi^{\prime}\left( \eta \right) - Pe\left\{ {\phi^{\prime\prime}\left( \eta \right)\left( {\psi \left( \eta \right) + {\Omega }} \right) + \psi^{\prime}\left( \eta \right)\phi^{\prime}\left( \eta \right)} \right\} = 0, $$

Subjected to the constraints:

16 $$ \begin{aligned} f\left( 0 \right) & = 0,\,\,f^{\prime}\left( \infty \right) = 0,\,\,\,f^{\prime}\left( 0 \right) = 1,\,\,\,g\left( 0 \right) = 0,\,\,\,\,\,g^{\prime}\left( 0 \right) = \alpha ,\,\,\,\,\,g^{\prime}\left( \infty \right) = 0, \\ \theta^{\prime}\left( 0 \right) & = - \frac{Bi}{{A_{4} }}\left( {1 - \theta \left( 0 \right)} \right),\,\,\,\,\theta \left( \infty \right) = 0,\,\,Nb\phi^{\prime}\left( 0 \right) + Nt\theta^{\prime}\left( 0 \right) = 0,\,\, \\ \,\phi \left( \infty \right) & = 0,\,\,\psi \left( 0 \right) = 1,\,\,\,\,\,\psi \left( \infty \right) = 0. \\ \end{aligned} $$

where

17 $$ \begin{aligned} A_{1} & = \mu_{nf} /\mu_{f} ,\quad A_{2} = \rho_{nf} /\rho_{f} ,\quad A_{3} = \sigma_{nf} /\sigma_{f} ,\quad A_{4} = k_{nf} /k_{f} ,\quad A_{5} = \left( {\rho C_{p} } \right)_{nf} /\left( {\rho C_{p} } \right)_{f} , \\ M & = \frac{{\sigma_{f} B_{0}^{2} }}{{\rho_{f} c}},\quad \alpha = \frac{b}{c},\quad Bi = \frac{{h_{f} }}{{k_{f} }}\sqrt {\frac{{\nu_{f} }}{c}} ,\quad Pe = \frac{{bW_{c} }}{{D_{m} }},\quad Nb = \frac{{\left( {\rho C_{p} } \right)_{np} }}{{\left( {\rho C_{p} } \right)_{f} }}\frac{{\delta D_{B} C_{\infty } }}{{\nu_{f} }},\quad \delta_{C} = c\lambda_{C} ,\quad K_{r} = \frac{{K_{1} }}{c}, \\ Nt & = \frac{{\left( {\rho C_{p} } \right)_{np} }}{{\left( {\rho C_{p} } \right)_{f} }}\frac{{D_{T} \left( {T_{f} - T_{\infty } } \right)}}{{T_{\infty } \nu_{f} }},\quad \delta_{T} = c\lambda_{T} ,\quad \Pr = \frac{{\mu_{f} \left( {C_{p} } \right)_{nf} }}{{k_{f} }},\quad Sc = \frac{{\nu_{f} }}{{D_{B} }},\quad E = \frac{{E_{a} }}{{kT_{\infty } }},\quad Lb = \frac{{\nu_{f} }}{{D_{m} }}. \\ \end{aligned} $$

Here, $M$ is magnetic parameter, $K$ is porosity factor, $\alpha$ is ratio factor, $Bi$ is thermal Biot number, $\Pr$ is Prandtl number, $\delta_{C}$ is mass relaxation time factor, $Nt$ is thermophoresis parameter, $E$ is activation energy factor, $\delta_{T}$ is thermal relaxation time factor, $Pe$ is Peclet number, $K_{r}$ is chemical reaction factor, $Nb$ is Brownian motion parameter, $Sc$ is Schmidt number and $Lb$ is Lewis number.

### Physical quantities of interest

This study centers on the key quantities skin friction, local Nusselt number, Sherwood number, and density number, which are formulated as:

18 $$ C_{fx} = \,\,\,\frac{{\tau_{w\,x} }}{{\,\rho_{f} \,\,u_{w}^{2} }}, $$

19 $$ C_{fy} = \frac{{\tau_{wy} }}{{\rho_{f} v_{w}^{2} }}, $$

20 $$ Nu_{x} = \frac{{q_{w} }}{{k_{f} \left( {T_{f} - T_{\infty } } \right)}}, $$

21 $$ Sh_{x} = \frac{{q_{m} }}{{D_{B} \left( {C_{w} - C_{\infty } } \right)}}, $$

22 $$ Nn_{x} = \frac{{q_{n} }}{{D_{m} \left( {N_{w} - N_{\infty } } \right)}}, $$

where

23 $$ \tau_{wx} = \mu_{nf} \left. {\frac{\partial u}{{\partial z}}} \right|_{z = 0} ,\,\,\,\tau_{wy} = \mu_{nf} \left. {\frac{\partial v}{{\partial z}}} \right|_{z = 0} ,\,\,\,q_{w} = - k_{nf} \left. {\frac{\partial T}{{\partial z}}} \right|_{z = 0} ,\,\,\,q_{m} = - D_{B} \left. {\frac{\partial C}{{\partial z}}} \right|_{z = 0} ,\,\,\,q_{n} = - D_{m} \left. {\frac{\partial N}{{\partial z}}} \right|_{z = 0} . $$

In this work there is no mass flux on surface of sheet i.e. $C_{w} = 0$ because of zero-mass flux conditions, therefore, Eq. (21) vanishes and Eqs. (18–20) and (22) altered as:

24 $$ C_{x} = A_{1} f^{\prime\prime}\left( 0 \right), $$

25 $$ C_{y} = A_{1} g^{\prime\prime}\left( 0 \right), $$

26 $$ Nu = - A_{4} \theta^{\prime}\left( 0 \right), $$

27 $$ Nn = - \psi^{\prime}\left( 0 \right), $$

where ${\mathrm{Re}}_{x} = \frac{{cx^{2} }}{{\nu_{f} }}$ and ${\mathrm{Re}}_{y} = \frac{{cy^{2} }}{{\nu_{f} }}$ are the local Reynolds numbers. Moreover, $C_{x} = \sqrt {{\mathrm{Re}}_{x} } C_{fx}$, $C_{y} = \sqrt {{\mathrm{Re}}_{y} } C_{fy}$, $Nu = \frac{{Nu_{x} }}{{\sqrt {{\mathrm{Re}}_{x} } }}$ and $Nn = \frac{{Nn_{x} }}{{\sqrt {{\mathrm{Re}}_{x} } }}$.

## Numerical solution

To solve the system defined by Eqs. (11–15), subject to the constraints of Eq. (16), the bvp4c numerical solver in MATLAB will be employed. This method is specifically designed for handling boundary value problems (BVPs) and provides a reliable approach for obtaining a numerical solution to the given set of coupled ODEs under the specified boundary conditions. This technique needs the conversion of higher order ODEs to 1st order ODEs. The computational domain is truncated to a finite interval with a uniform mesh grid containing 1 × 10^3^ equally spaced points. The numerical accuracy is controlled by prescribing the error tolerance of 1 × 10^–6^ to ensure table and accurate solution. The process is explained for current problem as:

28 $$ \begin{aligned} f & = \chi \left( 1 \right),\,\,\,f^{\prime} = \chi \left( 2 \right),\,f^{\prime\prime\prime} = \chi^{\prime}\left( 3 \right),\,\,\,f^{\prime\prime} = \chi \left( 3 \right),\, \\ g & = \chi \left( 4 \right),\,\,\,g^{\prime} = \chi \left( 5 \right),\,g^{\prime\prime\prime} = \chi^{\prime}\left( 6 \right),\,\,\,g^{\prime\prime} = \chi \left( 6 \right),\,\, \\ \theta & = \chi \left( 7 \right),\,\,\,\theta^{\prime} = \chi \left( 8 \right),\,\,\,\theta^{\prime\prime} = \chi^{\prime}\left( 8 \right), \\ \phi & = \chi \left( 9 \right),\,\,\,\phi^{\prime} = \chi \left( {10} \right),\,\,\,\phi^{\prime\prime} = \chi^{\prime}\left( {10} \right), \\ \psi & = \chi \left( {11} \right),\,\,\,\psi^{\prime} = \chi \left( {12} \right),\,\,\,\psi^{\prime\prime} = \chi^{\prime}\left( {12} \right), \\ \end{aligned} $$

Moreover,

29 $$ \chi^{\prime}\left( 3 \right) = - \frac{{A_{2} }}{{A_{1} }}\left\{ {\left( {\chi \left( 4 \right) + \chi \left( 1 \right)} \right)\chi \left( 3 \right) - \left( {\chi \left( 2 \right)} \right)^{2} - \frac{{A_{3} }}{{A_{2} }}M\chi \left( 2 \right) - \frac{{A_{1} }}{{A_{2} }}K\chi \left( 2 \right)} \right\}, $$

30 $$ \chi^{\prime}\left( 6 \right) = - \frac{{A_{2} }}{{A_{1} }}\left\{ {\left( {\chi \left( 4 \right) + \chi \left( 1 \right)} \right)\chi \left( 6 \right) - \left( {\chi \left( 5 \right)} \right)^{2} - \frac{{A_{3} }}{{A_{2} }}M\chi \left( 5 \right) - \frac{{A_{1} }}{{A_{2} }}K\chi \left( 5 \right)} \right\}, $$

31 $$ \chi^{\prime}\left( 8 \right) = - \frac{{\left\{ \begin{gathered} \Pr \left( {\chi \left( 4 \right) + \chi \left( 1 \right)} \right)\chi \left( 8 \right) + \frac{\Pr }{{A_{5} }}\left( {Nb\chi \left( {10} \right)\chi \left( 8 \right) + \left( {\chi \left( 8 \right)Nt} \right)^{2} } \right) \hfill \\ - \Pr \delta_{T} \left( {\chi \left( 1 \right) + \chi \left( 4 \right)} \right)\left( {\chi \left( 2 \right) + \chi \left( 5 \right)} \right)\chi \left( 8 \right) \hfill \\ \end{gathered} \right\}}}{{\left\{ {\frac{{A_{4} }}{{A_{5} }} - \Pr \delta_{T} \left( {\chi \left( 1 \right) + \chi \left( 4 \right)} \right)^{2} } \right\}}}, $$

32 $$ \chi^{\prime}\left( {10} \right) = - \frac{{\left\{ \begin{gathered} Sc\left( {\chi \left( 1 \right) + \chi \left( 4 \right)} \right)\chi \left( 8 \right) - K_{r} Sc\chi \left( 9 \right)\left( {1 + \sigma \chi \left( 7 \right)} \right)^{n} e^{{\left( { - \frac{E}{1 + \sigma \chi \left( 7 \right)}} \right)}} \hfill \\ - Sc\delta_{C} \left( {\chi \left( 4 \right) + \chi \left( 1 \right)} \right)\left( {\chi \left( 2 \right) + \chi \left( 5 \right)} \right)\chi \left( {10} \right) + \frac{Nt}{{Nb}}\chi^{\prime}\left( 8 \right) \hfill \\ \end{gathered} \right\}}}{{\left\{ {1 - Sc\delta_{C} \left( {\chi \left( 4 \right) + \chi \left( 1 \right)} \right)^{2} } \right\}}}, $$

33 $$ \chi^{\prime}\left( {12} \right) = - \left\{ {Lb\left( {\chi \left( 1 \right) + \chi \left( 4 \right)} \right)\chi \left( {12} \right) - Pe\left\{ {\chi^{\prime}\left( {10} \right)\left( {\chi \left( {11} \right) + {\Omega }} \right) + \chi \left( {12} \right)\chi \left( {10} \right)} \right\}} \right\}, $$

With boundary conditions:

34 $$ \begin{aligned} & \chi_{a} \left( 1 \right) = 0,\,\,\chi_{a} \left( 2 \right) = 1,\,\,\,\chi_{b} \left( 2 \right) = 0,\,\,\chi_{a} \left( 4 \right) = 0,\,\,\,\chi_{a} \left( 5 \right) = \alpha ,\,\,\,\,\,\chi_{b} \left( 5 \right) = 0, \\ & \chi_{a} \left( 8 \right) = - \frac{Bi}{{A_{4} }}\left( {1 - \chi_{a} \left( 7 \right)} \right),\,\,\, \\ & \chi_{b} \left( 7 \right) = 0, \\ & Nb\chi_{a} \left( {10} \right) + Nt\chi_{a} \left( 8 \right) = 0,\,\, \\ & \chi_{b} \left( 9 \right) = 0, \\ & \chi_{a} \left( {11} \right) = 1,\,\,\, \\ & \chi_{b} \left( {11} \right) = 0. \\ \end{aligned} $$

## Validation

To authenticate the current work its results are harmonized with results given in Ref. [41]. The comparison is conducted respectively in Tables 2 and 3 for $\left\{ { - f^{\prime\prime}\left( 0 \right)} \right\}$ and $\left\{ { - g^{\prime\prime}\left( 0 \right)} \right\}$ regarding variations in ratio factor $\left( \alpha \right)$ while keeping other parameters as zero. From both the tables it is obvious that there is a fine agreement amongst these results that guarantees the validation of current results.

**Table 2:** Validation of our results for $$- f^{\prime\prime}\left( 0 \right)$$ against $$\alpha$$ when $$M =$$$$K =$$$$\varphi_{1} = 0.0$$

$\alpha$	Reference [41] Results	Present results
0.00	1.00000	1.00000
0.20	1.039500	1.039510
0.40	1.074790	1.074793
0.60	1.109950	1.109944
0.80	1.142490	1.142491
1.00	1.173310	1.173312

**Table 3:** Validation of our results for $$- g^{\prime\prime}\left( 0 \right)$$ against $$\alpha$$ when $$M =$$$$K =$$$$\varphi_{1} = 0.0$$

$\alpha$	Reference [41] results	Current results
0.00	0.000000	0.000000
0.20	0.148740	0.1487412
0.40	0.349210	0.3492114
0.60	0.590630	0.5906323
0.80	0.866680	0.8666825
1.00	1.173710	1.1737112

## Discussion of results

This study investigates the steady MHD flow of a nanofluid on a porous medium, emphasizing advanced heat and mass transfer mechanisms. The mathematical model incorporates the combined effects of thermophoresis and Brownian motion to accurately describe nanoparticle behavior. It further analyzes the influence of Arrhenius activation energy on chemically reactive species. The thermal boundary layer is governed by a convective heating condition, while the concentration field is subjected to a physically realistic zero mass flux condition at the boundary. The bvp4c approach is used in this work to solve the modeled equations in dimension-free form. The default values of the embedded factors are chosen as: $\varphi = 0.04$, $d_{p} = 5.0$, $h = 1.5$, $K = 0.1$, $M = 1.0$, $\alpha = 0.1$, $Bi = 0.5$, $\Pr = 6.2$, $\delta_{C} = 0.1$, $Nt = 0.1$, $E = 1.0$, $Nb = 0.1$, $\delta_{T} = 0.1$, $Pe = 1.0$, $K_{r} = 0.1$, $Sc = 2.0$ and $Lb = 1.0$.

The effects of many parameters on different profiles are discussed in subsequent paragraphs:-

### Velocity profiles

**Fig. 2:**
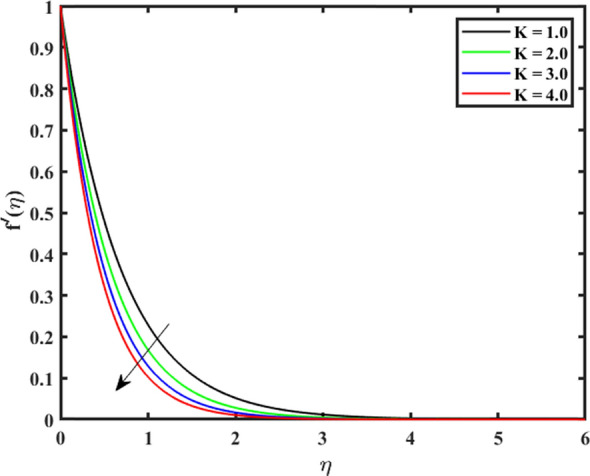
Impact of *K* on $f^{\prime}\left( \eta \right)$

**Fig. 3:**
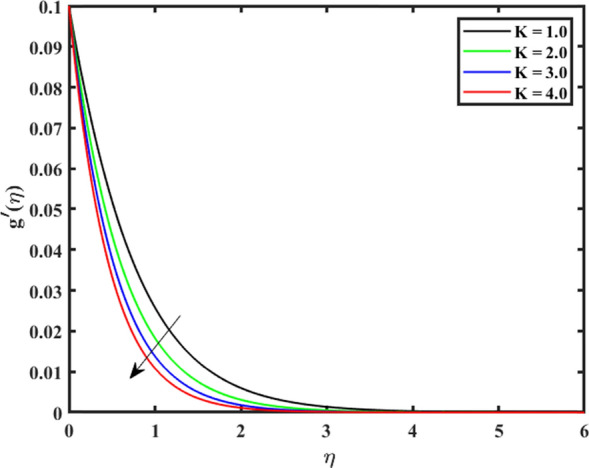
Impact of *K* on $g^{\prime}\left( \eta \right)$

**Fig. 4:**
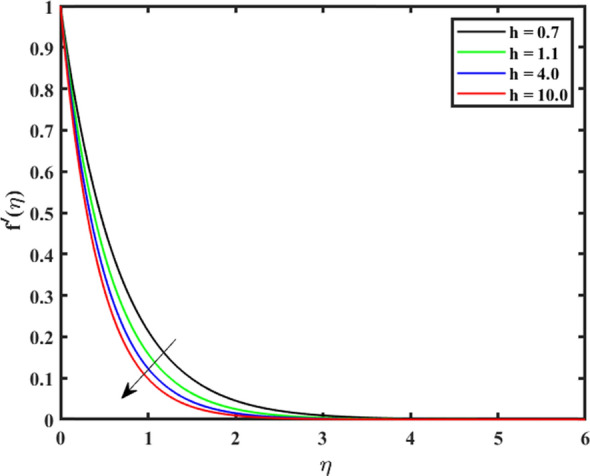
Impact of *h* on $f^{\prime}\left( \eta \right)$

**Fig. 5:**
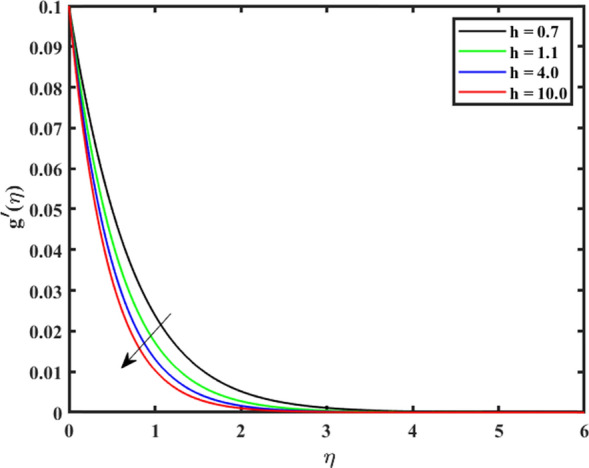
Impact of *h* on $g^{\prime}\left( \eta \right)$

**Fig. 6:**
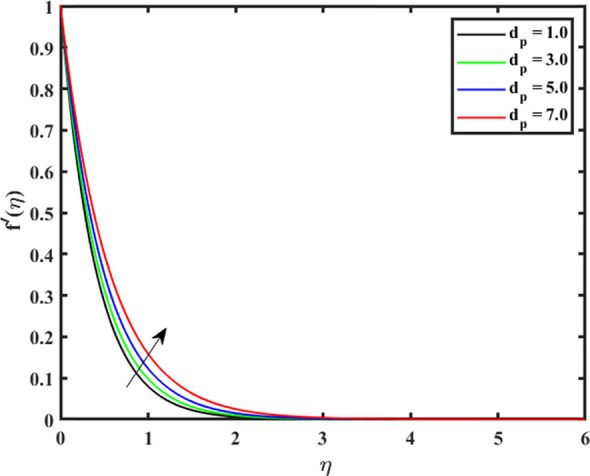
Impact of $d_{p}$ on $f^{\prime}\left( \eta \right)$

**Fig. 7:**
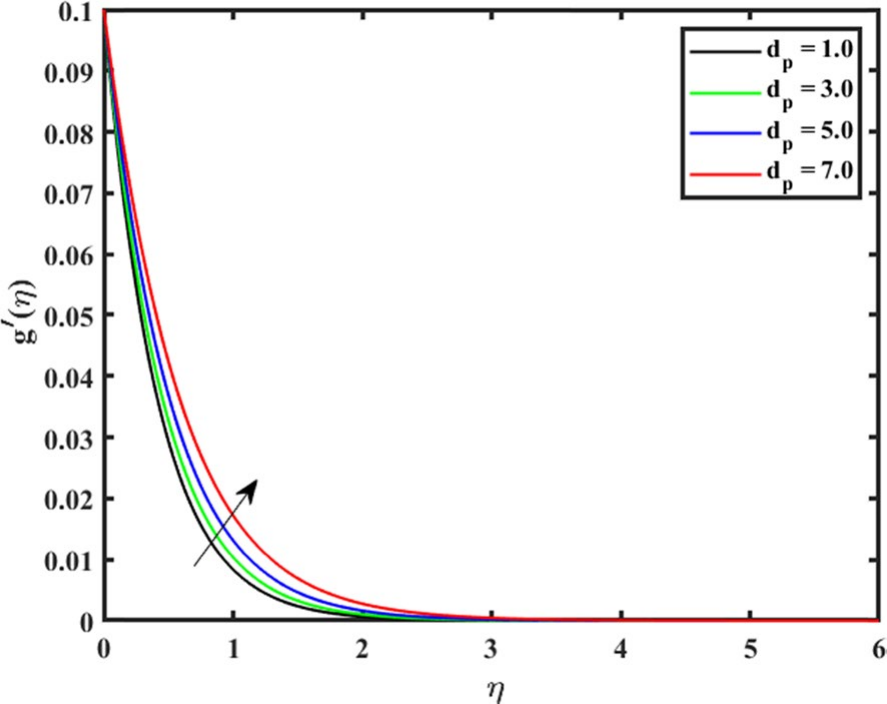
Impact of $d_{p}$ on $g^{\prime}\left( \eta \right)$

**Fig. 8:**
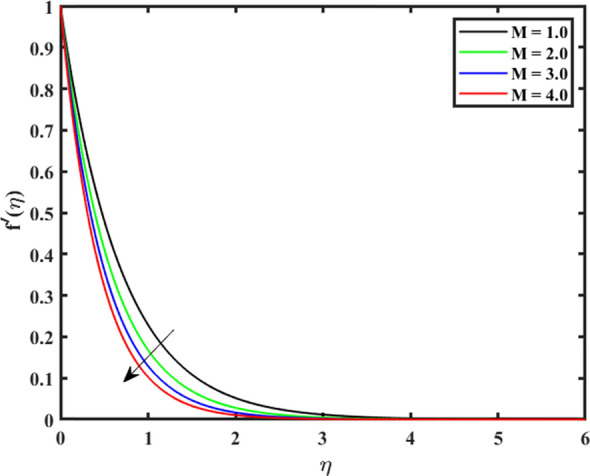
Impact of *M* on $f^{\prime}\left( \eta \right)$

**Fig. 9:**
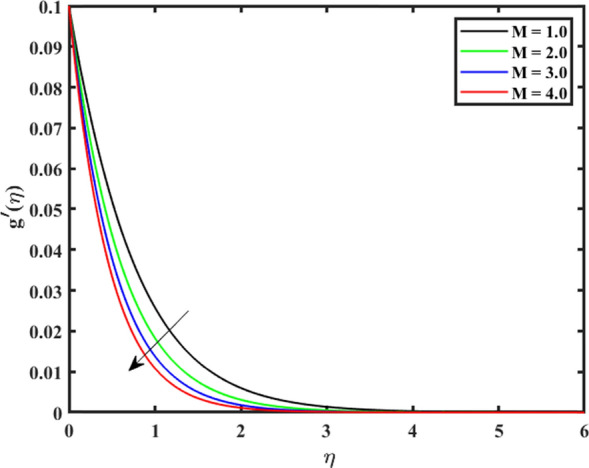
Impact of *M* on $g^{\prime}\left( \eta \right)$

The impression of numerous factors on primary velocity $\left\{ {f^{\prime}\left( \eta \right)} \right\}$ and secondary velocity $\left\{ {g^{\prime}\left( \eta \right)} \right\}$ is illustrated in Figs. 2, 3, 4, 5, 6, 7, 8 and 9. The impression of porosity factor (*K*) on $\left\{ {f^{\prime}\left( \eta \right)} \right\}$ and $\left\{ {g^{\prime}\left( \eta \right)} \right\}$ is examined in Figs. 2 and 3 with a declining behavior in both $\left\{ {f^{\prime}\left( \eta \right)} \right\}$ and $\left\{ {g^{\prime}\left( \eta \right)} \right\}$ for growth in (*K*). The observed decline in $\left\{ {f^{\prime}\left( \eta \right)} \right\}$ with an increasing (*K*) is a direct consequence of heightened flow resistance within the porous medium. As (*K*) rises, it typically corresponds to a lower permeability, meaning the surface offers more frictional drag against the fluid flow. This intensified resistive force directly opposes the primary direction of motion, absorbing momentum from the mainstream flow. Consequently, the fluid’s axial momentum is progressively dissipated, leading to a substantial retardation of $\left\{ {f^{\prime}\left( \eta \right)} \right\}$ across the boundary layer as explained in Fig. 2. The suppression of $\left\{ {g^{\prime}\left( \eta \right)} \right\}$ with increased porosity is attributed to the dominant dampening effect of the porous structure. Any transverse motion induced by external forces like rotation or magnetic fields is strongly impeded by the dense matrix. The porous medium acts as a distributed frictional sink for momentum in all directions, but its impact is particularly noticeable on the typically weaker secondary flows. The enhanced resistance restricts the fluid’s ability to develop or sustain cross-stream components, effectively damping out secondary circulations and confining the flow more strictly to the primary direction as explained in Fig. 3. The impression of inter-particles spacing (*h*) on $\left\{ {f^{\prime}\left( \eta \right)} \right\}$ and $\left\{ {g^{\prime}\left( \eta \right)} \right\}$ is examined in Figs. 4 and 5 with a declining behavior in both $\left\{ {f^{\prime}\left( \eta \right)} \right\}$ and $\left\{ {g^{\prime}\left( \eta \right)} \right\}$ for growth in (*h*). The decline in $\left\{ {f^{\prime}\left( \eta \right)} \right\}$ with increased (*h*) can be physically interpreted through its inverse relationship with the effective viscosity and microscopic drag within the nanofluid. While greater spacing reduces direct particle collisions, it simultaneously allows for more significant velocity gradients and relative motion between the fluid layers and the suspended nanoparticles, especially under shear. This enhanced slip velocity and the consequent rise in momentum diffusion resistance raise the effective viscosity of the suspension. As a result, the dominant viscous damping force within the boundary layer strengthens, absorbing more momentum from the primary flow direction. Therefore, the mainstream axial flow experiences greater resistance as examined in Fig. 4, leading to its systematic retardation as the particle spacing increases, even in the absence of a denser particle matrix. The reduction in $\left\{ {g^{\prime}\left( \eta \right)} \right\}$ with larger (*h*) is attributed to the weakened micro-scale perturbations and vortex-inducing mechanisms caused by nanoparticle interactions. Closely spaced particles generate localized disturbances, micro-rotations, and asymmetric drag forces that can induce and sustain transverse flow components. As the spacing increases, this particle-induced micro-convection and rotational momentum transfer diminish significantly. The flow becomes more homogenized, and the ability of discrete nanoparticles to generate or amplify secondary circulations (often driven by external fields like magnetism or rotation) is severely compromised. Consequently, the dampening effect of the base fluid’s viscosity dominates, suppressing the development and intensity of any cross-stream or swirling motions, thereby reducing the secondary velocity profiles $\left\{ {g^{\prime}\left( \eta \right)} \right\}$ as revealed in Fig. 5. The impression of nanoparticles’ radius $\left( {d_{p} } \right)$ on $\left\{ {f^{\prime}\left( \eta \right)} \right\}$ and $\left\{ {g^{\prime}\left( \eta \right)} \right\}$ is examined in Figs. 6 and 7 with a declining behavior in both $\left\{ {f^{\prime}\left( \eta \right)} \right\}$ and $\left\{ {g^{\prime}\left( \eta \right)} \right\}$ for growth in $\left( {d_{p} } \right)$. The observed decline in $\left\{ {f^{\prime}\left( \eta \right)} \right\}$ with growing $\left( {d_{p} } \right)$ can be attributed to a substantial intensification in effective viscosity and fluid-particle drag. Larger nanoparticles possess a greater cross-sectional area, which substantially amplifies the viscous resistance and momentum damping within the flow. This enhanced resistance is particularly dominant in the primary direction, where the bulk motion is directly opposed by the increased frictional interaction between the fluid and the enlarged particle surfaces. Consequently, the energy required to sustain the primary flow through the nanofluid increases, causing a systematic retardation of the axial velocity $\left\{ {f^{\prime}\left( \eta \right)} \right\}$ across the boundary layer as examined in Fig. 6. The reduction in $\left\{ {g^{\prime}\left( \eta \right)} \right\}$ with larger $\left\{ {g^{\prime}\left( \eta \right)} \right\}$ stems from the suppression of particle-induced micro-mixing and cross-flow momentum generation. While larger particles have greater inertia, their reduced ability to follow the transverse fluctuations of the fluid due to increased Stokes drag dampens the energy transfer into secondary flows. The strengthened viscous dissipation associated with larger particles also effectively absorbs the kinetic energy of any developing vortices or lateral motions. Consequently, the secondary flow structures are weakened, and the fluid’s capacity to develop and sustain velocity components orthogonal to the primary flow direction is notably diminished as examined in Fig. 7. The impressions of magnetic parameter $\left( M \right)$ on $\left\{ {f^{\prime}\left( \eta \right)} \right\}$ and $\left\{ {g^{\prime}\left( \eta \right)} \right\}$ are examined in Figs. 8 and 9 with a declining behavior in both $\left\{ {f^{\prime}\left( \eta \right)} \right\}$ and $\left\{ {g^{\prime}\left( \eta \right)} \right\}$ for growth in (*M*). Physically, as (*M*) grows, the intensity of opposing Lorentz force increases, thereby dissipating kinetic energy from the mainstream flow. This results in the momentum layer at borderline becoming thicker, with a corresponding retardation of the axial velocity profile $\left\{ {f^{\prime}\left( \eta \right)} \right\}$, effectively suppressing the primary flow as explained in Fig. 8. The reduction in $\left\{ {g^{\prime}\left( \eta \right)} \right\}$ with an elevated (*M*) is due to the magnetic damping of vortices and lateral fluid motion. The Lorentz force resists not only the primary flow but also any velocity component perpendicular to the magnetic field lines. This force suppresses fluctuations and instabilities that typically generate and sustain secondary flows. Consequently, the magnetic field stabilizes the boundary layer, inhibiting the formation and intensity of cross-stream velocity components. This leads to a damping of vertical structures and a substantial decline of secondary velocity profiles $\left\{ {g^{\prime}\left( \eta \right)} \right\}$ as explained in Fig. 9.

### Temperature profiles

**Fig. 10:**
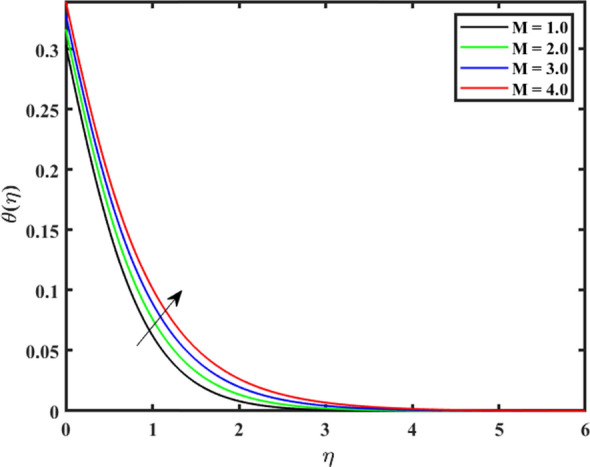
Impact of *M* on $\theta \left( \eta \right)$

**Fig. 11:**
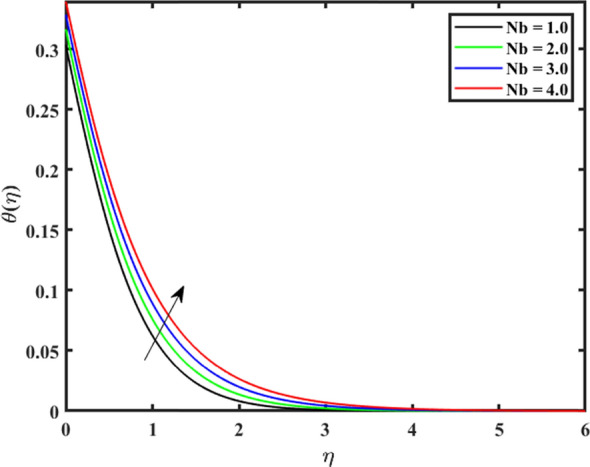
Impact of $Nb$ on $\theta \left( \eta \right)$

**Fig. 12:**
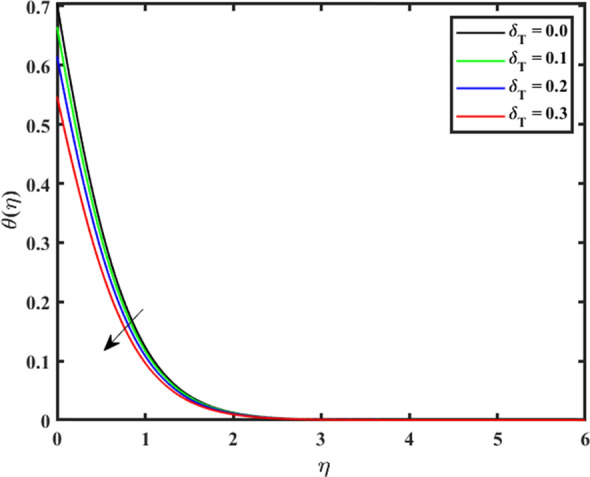
Impact of $\delta_{T}$ on $\theta \left( \eta \right)$

**Fig. 13:**
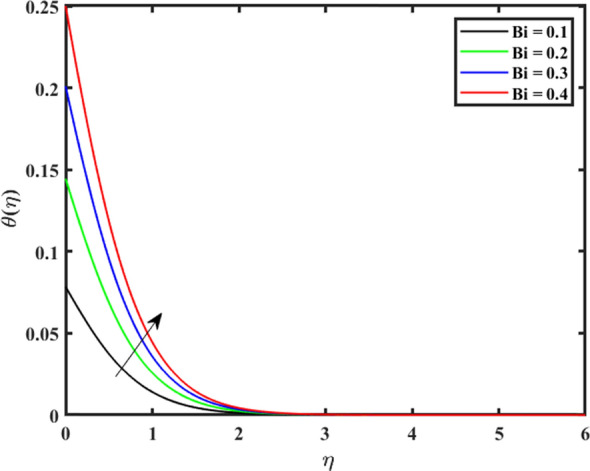
Impact of $Bi$ on $\theta \left( \eta \right)$

**Fig. 14:**
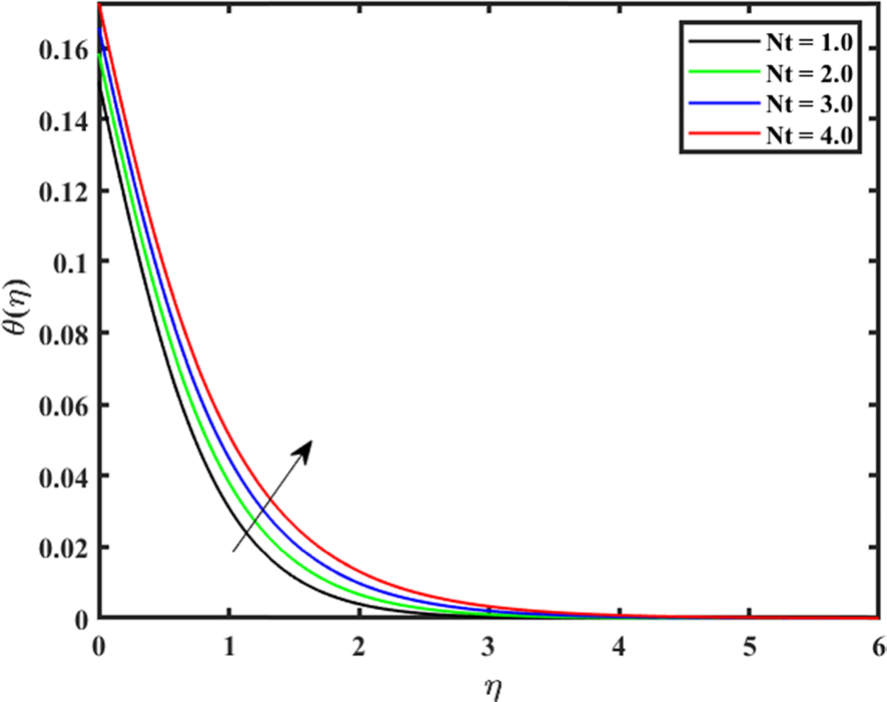
Impact of $Nt$ on $\theta \left( \eta \right)$

The impression of numerous factors on thermal profiles $\left\{ {\theta \left( \eta \right)} \right\}$ is illustrated in Figs. 10, 11, 12, 13 and 14. The impression of (*M*) on $\left\{ {\theta \left( \eta \right)} \right\}$ is examined in Fig. 10 with an augmenting behavior in $\left\{ {\theta \left( \eta \right)} \right\}$ for growth in (*M*). The observed growth in $\left\{ {\theta \left( \eta \right)} \right\}$ with an intensification in (*M*) is primarily attributed to the enhanced magnetic viscous dissipation within the electrically conductive fluid. As (*M*) rises the Lorentz force opposing the fluid motion intensifies. This opposition does more than just retard the flow; it translates a substantial portion of the fluid’s kinetic to inner thermal energy through frictional (viscous) dissipation. Simultaneously, the electric currents induced by the motion of the conductive fluid inside magnetic field encounter resistance, generating additional volumetric heating known as Ohmic or Joule heating. This dual effect acts as a distributed internal heat source throughout the boundary layer. Furthermore, the magnetic damping decreases the convective cooling rate and allows heat to accumulate and diffuse more effectively. Consequently, despite the reduction in velocity, the net thermal energy within the system rises, leading to an elevation in the temperature distribution across the flow domain. The impression of Brownian motion factor (*Nb*) on $\left\{ {\theta \left( \eta \right)} \right\}$ is examined in Fig. 11 with an augmenting behavior in $\left\{ {\theta \left( \eta \right)} \right\}$ for growth in (*Nb*). The rise in $\left\{ {\theta \left( \eta \right)} \right\}$ with a growing (*Nb*) is a direct consequence of enhanced microscopic energy transport within the nanofluid. Physically, as (*Nb*) increases, nanoparticles exhibit more vigorous and frequent collisions and interactions with the surrounding fluid molecules. This intensified microscopic activity significantly augments two key phenomena: first, it facilitates a more efficient interfacial heat transfer amid the nanoparticles and fluid through direct kinetic energy exchange; second, it promotes a greater degree of micro-scale fluid mixing, which enhances the thermal dispersion of energy throughout the boundary layer. This internal mixing effectively reduces the fluid’s thermal resistance and rises its effective thermal conductance. Consequently, even without altering the external heat input, the thermal energy is distributed more rapidly and uniformly, leading to a higher overall temperature distribution and a noticeable thickening and intensification of the thermal layer at boundary. The impression of thermal relaxation time factor $\left( {\delta_{T} } \right)$ on $\left\{ {\theta \left( \eta \right)} \right\}$ is examined in Fig. 12 with a declining behavior in $\left\{ {\theta \left( \eta \right)} \right\}$ for growth in $\left( {\delta_{T} } \right)$. The observed decline in $\left\{ {\theta \left( \eta \right)} \right\}$ with a rise in $\left( {\delta_{T} } \right)$ is a direct manifestation of a retarded heat transfer mechanism. This factor, central to the Cattaneo–Christov heat flux model, represents the finite time required for the material to establish a thermal equilibrium after a temperature gradient is applied. A larger $\left( {\delta_{T} } \right)$ signifies that the propagation of thermal signals is delayed, meaning the diffusion of heat cannot instantaneously follow the imposed temperature gradients. Consequently, the non-Fourier thermal lag effect becomes more noticeable, causing the heat flux to respond more slowly to changes in the thermal field. This introduces a phase lag that disrupts the immediate and full transmission of thermal energy through the medium, effectively dampening the temperature response. Consequently, heat accumulates less efficiently that lowers the profiles $\left\{ {\theta \left( \eta \right)} \right\}$. The impression of thermal Biot number (*Bi*) on $\left\{ {\theta \left( \eta \right)} \right\}$ is examined in Fig. 13 with a surging behavior in $\left\{ {\theta \left( \eta \right)} \right\}$ for growth in (*Bi*). The augmentation in $\left\{ {\theta \left( \eta \right)} \right\}$ with an intensification in (*Bi*) is fundamentally linked to a shift in thermal resistance dominance. Physically, low (*Bi*) indicates that internal resistance is negligible, causing accumulation of more temperature on the surface of sheet. Conversely, as (*Bi*) grows, the internal conductive resistance becomes more significant relative to the convective cooling at the boundary. This means the surface heat transfer via convection from the adjacent fluid occurs more rapidly than the solid can conduct heat away from its interior. Consequently, a steeper temperature gradient develops within the material to drive the necessary conductive heat flux to the surface, causing a noticeable increase in the internal temperature profile. In the context of a convective boundary condition, a higher (*Bi*) physically corresponds to a stronger thermal interaction with the surrounding medium, which supplies more thermal energy to the system, thereby elevating the overall temperature distribution. The impression of thermophoretic factor (*Nt*) on $\left\{ {\theta \left( \eta \right)} \right\}$ is examined in Fig. 14 with a surging behavior in $\left\{ {\theta \left( \eta \right)} \right\}$ for growth in (*Nt*). The augmentation in $\left\{ {\theta \left( \eta \right)} \right\}$ with an escalation in (*Nt*) arises from the direct coupling between temperature gradients and nanoparticle migration. The thermophoresis factor (*Nt*) quantifies the intensity of the thermophoretic force, which drives nanoparticles from hotter regions toward cooler inside fluid. As this factor rises, nanoparticles migrate more promptly and accumulate in the cooler parts of the boundary layer, typically near the surface. This redistribution significantly modifies the local thermophysical properties of the nanofluid, particularly enhancing the effective thermal conductivities in the vicinity of the wall due to the higher nanoparticle concentration. The resulting improved conductive heat transfer in this region reduces the thermal resistance near the boundary, enabling more efficient heat absorption from the heated surface. Consequently, this process elevates the temperature distribution across the entire thermal layer at boundary, leading to a noticeable thickening and intensification of the thermal profile.

### Concentration profiles

**Fig. 15:**
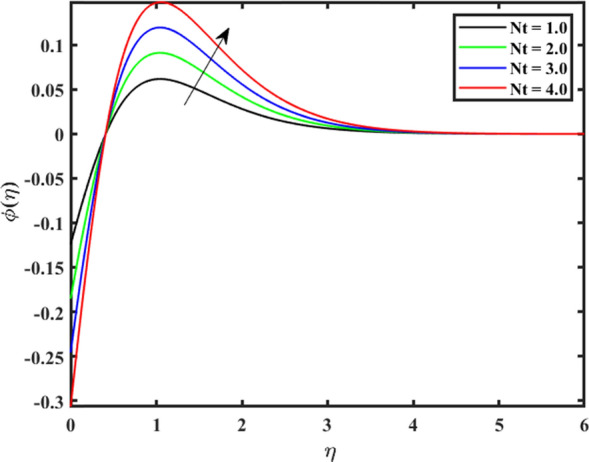
Impact of $Nt$ on $\phi \left( \eta \right)$

**Fig. 16:**
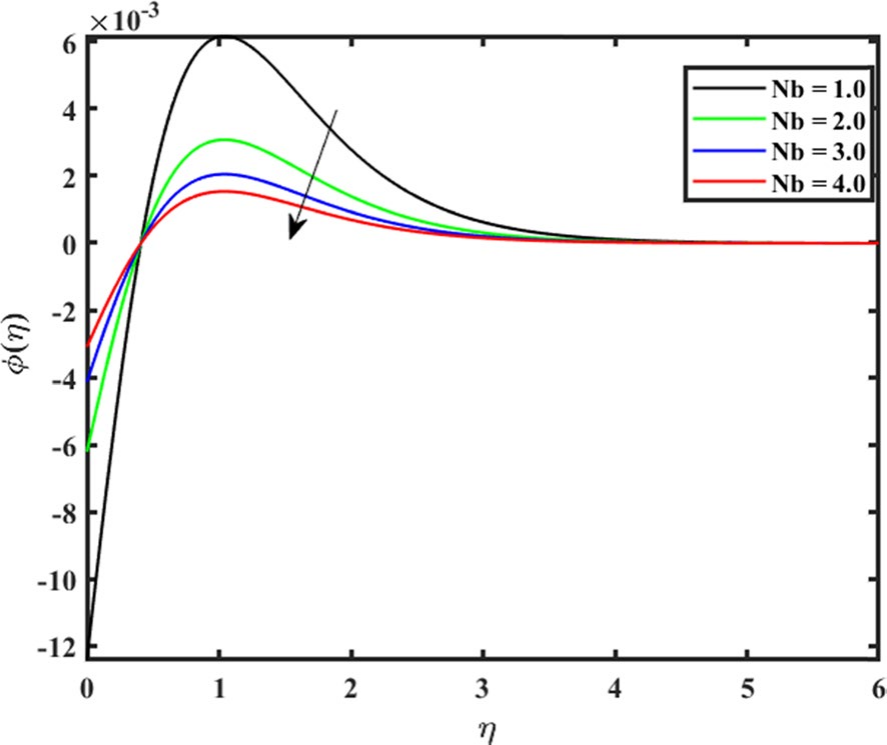
Impact of $Nb$ on $\phi \left( \eta \right)$

**Fig. 17:**
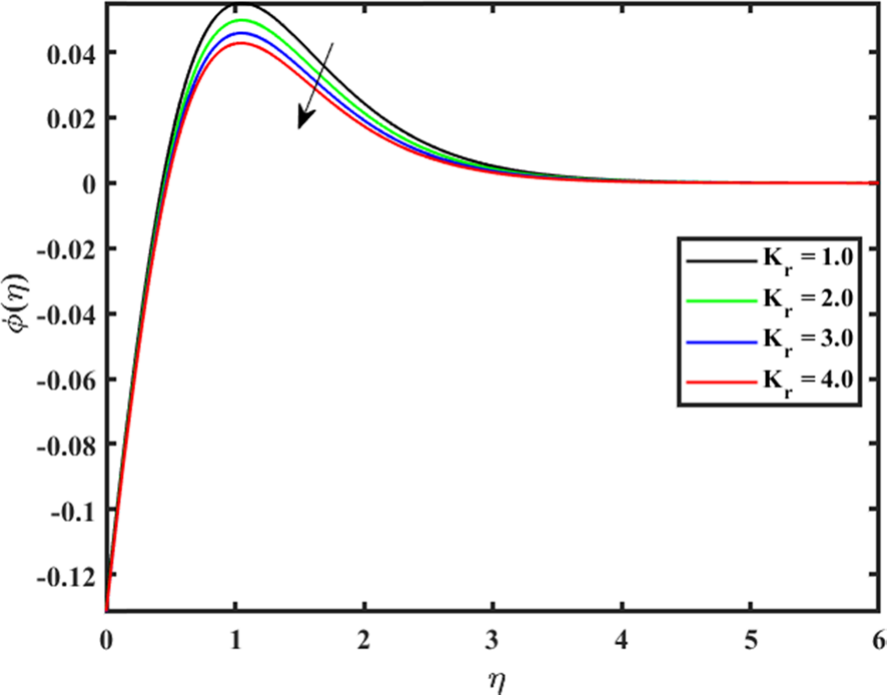
Impact of $K_{r}$ on $\phi \left( \eta \right)$

**Fig. 18:**
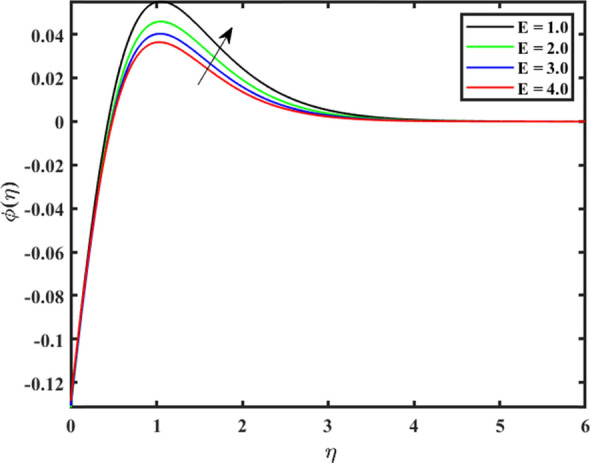
Impact of $E$ on $\phi \left( \eta \right)$

**Fig. 19:**
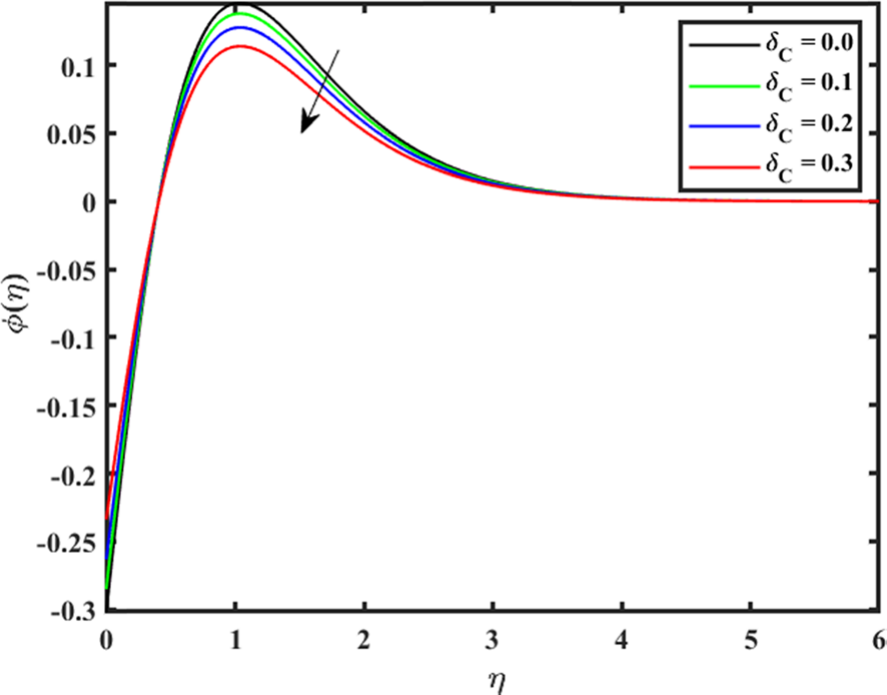
Impact of $\delta_{C}$ on $\phi \left( \eta \right)$

**Fig. 20:**
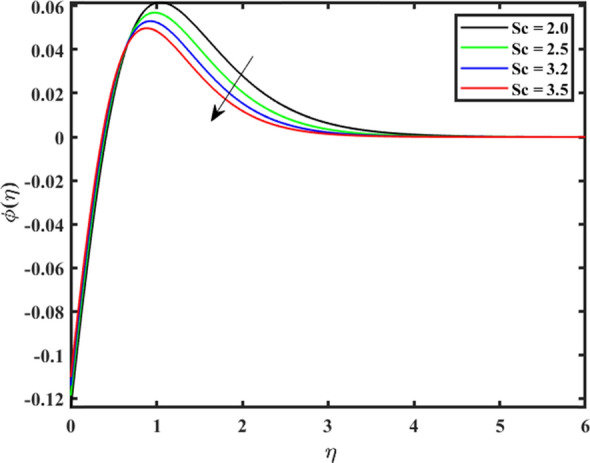
Impact of $Sc$ on $\phi \left( \eta \right)$

The impression of many factors on concentration panels $\left\{ {\phi \left( \eta \right)} \right\}$ is illustrated in Figs. 15, 16, 17, 18, 19 and 20. The impression of thermophoresis factor (*Nt*) on $\left\{ {\phi \left( \eta \right)} \right\}$ is examined in Fig. 15 with an augmenting behavior of $\left\{ {\phi \left( \eta \right)} \right\}$ for growth in (*Nt*). The augmentation in $\left\{ {\phi \left( \eta \right)} \right\}$ with an intensification in (*Nt*) is a direct outcome of the intensified thermophoretic migration driven by the applied thermal gradient. The thermophoresis factor represents the strength of the particle flux induced by a temperature gradient, causing nanoparticles to move from zones of higher temperature to cooler zones. As this factor grows, the thermophoretic velocity of the nanoparticles increases substantially, leading to a more noticeable and rapid accumulation of particles in the cooler areas of the boundary layer, typically adjacent to the surface. This creates a sharper concentration gradient as particles are effectively pushed and concentrated near the wall, while being depleted from the hotter mainstream region. Consequently, the local nanoparticle volume fraction rises significantly in the vicinity of the boundary, resulting in a steeper, more noticeable concentration profile and a noticeable thickening of the solutal boundary layer as thermophoresis dominates over the dispersive effects of Brownian diffusion. The impression of (*Nb*) on $\left\{ {\phi \left( \eta \right)} \right\}$ is examined in Fig. 16 with a reducing behavior of $\left\{ {\phi \left( \eta \right)} \right\}$ for growth in (*Nb*). The reduction in $\left\{ {\phi \left( \eta \right)} \right\}$ with an intensification in (*Nb*) is a direct consequence of enhanced microscopic diffusion and dispersion. The factor (*Nb*) quantifies the intensity of random, chaotic nanoparticle movement driven by thermal energy. As this factor grows, particles experience more vigorous and frequent collisions, leading to stronger dispersive mixing throughout the fluid domain. This heightened random motion acts against the formation of localized particle accumulation, effectively smoothing out a sharp concentration gradients. In particular, Brownian diffusion counteracts and dominates over directional mechanisms like thermophoresis that tend to concentrate particles in specific regions (e.g., near a cooler wall). Consequently, the nanoparticles become more uniformly distributed across the boundary layer, lowering the peak concentration near the surface and flattening the overall concentration profile. This results in a thinner and less noticeable solutal boundary layer as enhanced diffusion promotes uniformity rather than segregation. The impression of chemical reactivity factor $\left( {K_{r} } \right)$ on $\left\{ {\phi \left( \eta \right)} \right\}$ is examined in Fig. 17 with a reducing behavior of $\left\{ {\phi \left( \eta \right)} \right\}$ for growth in (*Nb*). The reduction in $\left\{ {\phi \left( \eta \right)} \right\}$ with a rise in $\left( {K_{r} } \right)$ is a direct consequence of the enhanced depletion of reactive species. The factor $\left( {K_{r} } \right)$ represents the rate at which chemical reactions occur typically modeled as a first-order or higher-order homogeneous reaction. As this factor grows, the reaction rate intensifies, leading to a more rapid conversion of the diffusing species as they transport through the fluid medium. This effectively acts as a distributed volumetric sink for concentration within the solutal boundary layer. The heightened reaction process competes directly with the diffusion and convection mechanisms, removing mass from the system faster than it can be replenished by the boundary conditions. Consequently, the concentration differences close to surface becomes steeper as species are depleted more quickly, causing a lower overall concentration distribution and a thinning of the concentration layer at borderline as chemical consumption outweighs mass transport. The impression of chemical reactivity factor (*E*) on $\left\{ {\phi \left( \eta \right)} \right\}$ is examined in Fig. 18 with a rising behavior of $\left\{ {\phi \left( \eta \right)} \right\}$ for growth in (*E*). The augmentation in $\left\{ {\phi \left( \eta \right)} \right\}$ with an increase in (*E*) stems from its inhibitory effect on the rate of chemical consumption. Activation energy represents the lowest energy threshold mandatory for a chemical reaction to proceed. A higher (*E*) makes this energy barrier more significant, thereby slowing the reaction kinetics and dropping the rate at which the diffusing species are consumed within the boundary layer. This diminished reactivity effectively weakens the volumetric sink term in the mass transfer equation, allowing species to accumulate rather than being rapidly depleted. Consequently, both molecular and nanoparticle diffusion processes become more dominant relative to chemical destruction, enabling a greater penetration and higher concentration of species throughout the domain. Consequently, the solutal boundary layer thickens, and the overall concentration panels are elevated, as the retarded chemical reaction permits mass to build up rather than be quickly converted. The impression of mass relaxation time factor $\left( {\delta_{C} } \right)$ on $\left\{ {\phi \left( \eta \right)} \right\}$ is examined in Fig. 19 with a rising behavior of $\left\{ {\phi \left( \eta \right)} \right\}$ for growth in $\left( {\delta_{C} } \right)$. The reduction in $\left\{ {\phi \left( \eta \right)} \right\}$ with a rise in $\left( {\delta_{C} } \right)$ is fundamentally a consequence of retarded or non-Fickian mass diffusion, where a finite time lag is introduced between the imposition of a concentration gradient and the resulting mass flux. This relaxation time represents the delay required for the diffusing species to respond to changes in the concentration field, effectively slowing the transport process. A larger mass relaxation time means that fluid take longer to migrate, which suppresses the rate at which mass can accumulate within the boundary layer. This delayed response diminishes the effective diffusivity and weakens the overall mass transfer, thereby lowering the concentration distribution. In essence, the increased relaxation time acts as an internal temporal resistance, reducing the efficiency of mass penetration and leading to a flatter and more depleted concentration profile. The impression of Schmidt number (*Sc*) on $\left\{ {\phi \left( \eta \right)} \right\}$ is examined in Fig. 20 with a declining behavior of $\left\{ {\phi \left( \eta \right)} \right\}$ for growth in (*Sc*). The reduction in $\left\{ {\phi \left( \eta \right)} \right\}$ with an intensification in (*Sc*) is fundamentally tied to the relative domination of momentum dissemination over mass diffusion. A higher (*Sc*) signifies that mass diffuses significantly slower than momentum, indicating a thicker viscous boundary layer and a thinner concentration layer at boundary. As this number grows, mass transfer becomes increasingly diffusion-limited, meaning species cannot penetrate as deeply into the flow domain and are confined to a narrower region near the boundary that ultimately declined $\left\{ {\phi \left( \eta \right)} \right\}$.

### Microorganisms profiles

**Fig. 21:**
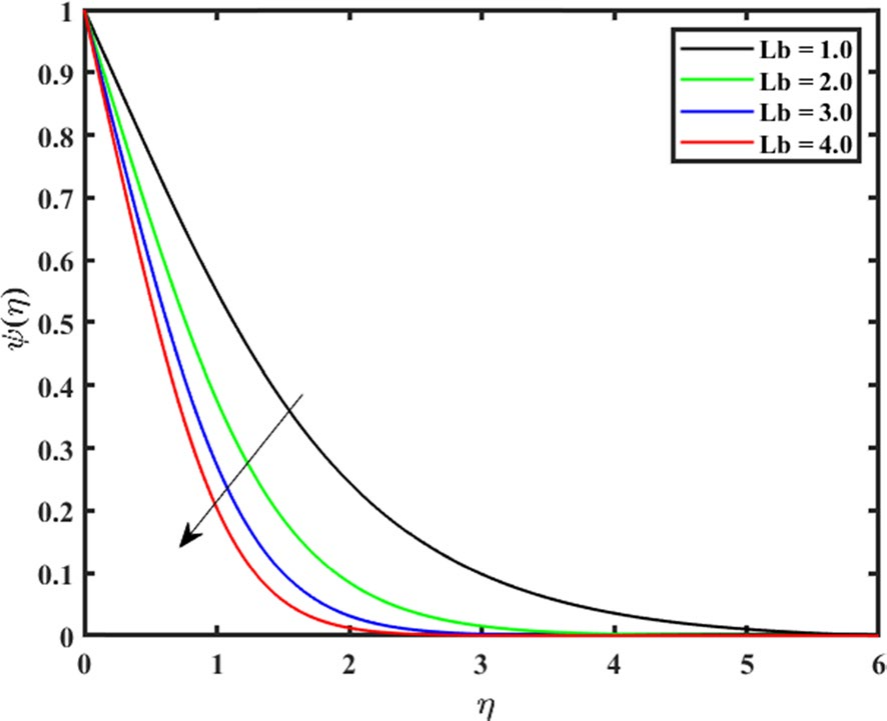
Impact of $Lb$ on $\psi \left( \eta \right)$

**Fig. 22:**
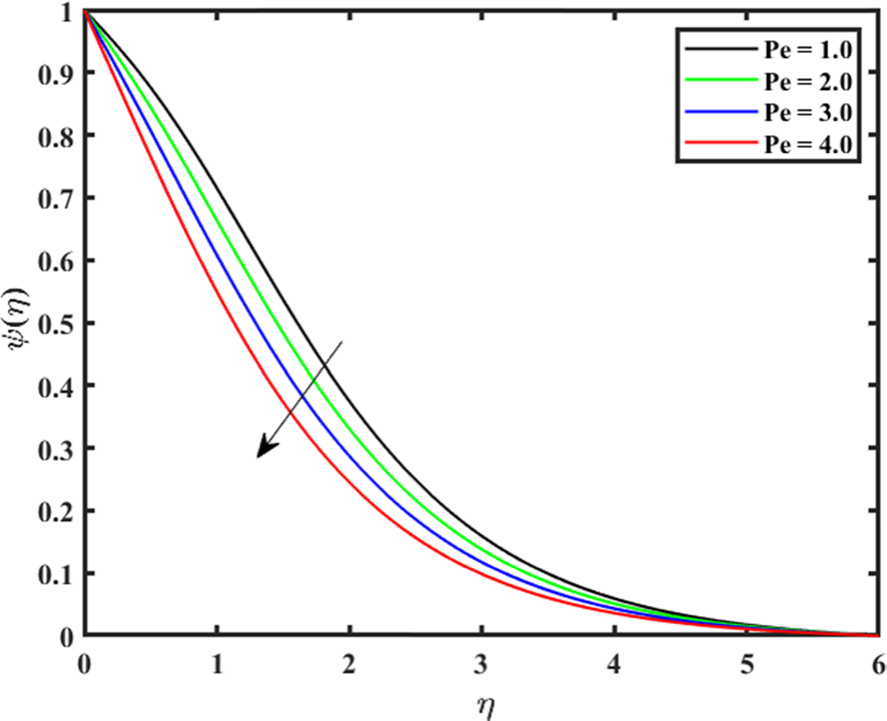
Impact of $Pe$ on $\psi \left( \eta \right)$

The impression of numerous factors on microorganisms profiles $\left\{ {\psi \left( \eta \right)} \right\}$ is illustrated in Figs. 21 and 22. The impression of bio-convection Lewis number (*Lb*) on $\left\{ {\psi \left( \eta \right)} \right\}$ is examined in Fig. 21 with declining behavior of $\left\{ {\psi \left( \eta \right)} \right\}$ for growth in (*Nt*). The observed reduction in $\left\{ {\psi \left( \eta \right)} \right\}$ with an intensification in (*Lb*) is fundamentally tied to the sharpening competition between viscous diffusion and microbial motility. Physically, a higher (*Lb*) signifies that the diffusion of microorganisms is substantially slower than the viscous diffusion of momentum. This implies that the hydrodynamic layer thickens at boundary, more readily than the microorganism concentration boundary layer, confining microbial motion to an increasingly narrow region near the surface. As this number grows, the effective transport of microorganisms away from the surface becomes more diffusion-limited and less efficient, meaning the swimming and convective redistribution of microbes cannot overcome the growing resistance to their spread. Consequently, microorganisms accumulate more densely at the boundary but experience a steeper concentration decay and a thinner active layer, leading to an overall suppression and reduction of the microorganism concentration profile $\left\{ {\psi \left( \eta \right)} \right\}$ across the domain. The effect of Peclet number (*Pe*) on $\left\{ {\psi \left( \eta \right)} \right\}$ is examined in Fig. 22 with declining behavior of $\left\{ {\psi \left( \eta \right)} \right\}$ for growth in (*Pe*). The reduction in $\left\{ {\psi \left( \eta \right)} \right\}$ with rise in (*Pe*) is a direct outcome of the shift in transport dominance from microbial diffusion to fluid convection. Physically, higher (*Pe*) specifies that advection driven by the bulk fluid motion becomes significantly stronger than the random, self-propelled diffusion of microorganisms. In this regime, the swift convective flow effectively sweeps microorganisms along the primary flow direction, limiting their ability to disperse laterally and accumulate within the boundary layer. This enhanced advection also delays the microbes’ natural upward swimming (in bio-convection) from counteracting the flow, as their directional motility is overpowered by the intensified mainstream velocity. Consequently, the residence time of microorganisms near the surface is reduced, thinning the microbial layer at borderline and leading to a more rapid depletion of microorganism concentration away from the boundary, thereby flattening and suppressing the overall microorganism profile.

### Table discussion

Table 1 examines the thermal and physical characteristics of Au and water. Tables 2 and 3 illustrate comparative study of current work. Tables 4 and 5 explains the impression of various factors on $\left\{ {\sqrt {{\mathrm{Re}}_{x} } C_{fx} } \right\}$ along x-axis and $\left\{ {\sqrt {{\mathrm{Re}}_{y} } C_{fy} } \right\}$ along y-axis. The augmentation in $\left\{ {\sqrt {{\mathrm{Re}}_{x} } C_{fx} } \right\}$ and $\left\{ {\sqrt {{\mathrm{Re}}_{y} } C_{fy} } \right\}$ with increasing $\left( \alpha \right)\,$, (*M*) and (*K*) stems from a synergistic intensification of resistive forces within the boundary layer. An elevated $\left( \alpha \right)\,$ enhances Coriolis forces, which induce additional cross-flow velocity components and viscous shear stress. Higher (*M*) strengthens the Lorentz force, producing a direct resistive drag that impedes flow, thereby increasing the velocity gradient at the wall. Simultaneously, a larger (*K*) reduces permeability, amplifying Darcy drag from the porous matrix and further resisting fluid motion. These combined forces Coriolis, magnetic, and Darcy resistance collectively thicken the momentum boundary layer while sharpening the velocity gradients at the surface. This results in a significant rise in shear stress along both the primary (x-axis) and secondary (y-axis) directions, manifesting as a marked increase in skin friction coefficients. Table 6 explains the impression of numerous parameters on Nusselt number $\left\{ {Nu_{x} \left( {{\mathrm{Re}}_{x} } \right)^{ - 1/2} } \right\}$. With progression in Brownian motion parameter and thermal Biot number there is growth in $\left\{ {Nu_{x} \left( {{\mathrm{Re}}_{x} } \right)^{ - 1/2} } \right\}$ while with augmentation in thermophoresis factor and thermal relaxation time parameter there is drop in $\left\{ {Nu_{x} \left( {{\mathrm{Re}}_{x} } \right)^{ - 1/2} } \right\}$. The opposing trends in $\left\{ {Nu_{x} \left( {{\mathrm{Re}}_{x} } \right)^{ - 1/2} } \right\}$ arise from distinct mechanisms influencing the wall temperature gradient. An increase in the thermal Biot number signifies stronger convective heating from the surrounding fluid, which steepens the temperature gradient at the boundary, thereby enhancing conductive heat transfer and raising $\left\{ {Nu_{x} \left( {{\mathrm{Re}}_{x} } \right)^{ - 1/2} } \right\}$. Similarly, a higher Brownian motion factor intensifies microscale particle–fluid interactions and thermal dispersion, effectively improving the nanofluid’s thermal conductance and promoting a steeper thermal gradient at the wall. In contrast, an augmented thermophoresis factor drives nanoparticles away from the heated surface toward cooler regions, depleting the particle concentration and thus the local thermal conductivity enhancement near the wall, which weakens the temperature gradient and reduces heat transfer. Likewise, an increase in the thermal relaxation time factor introduces a phase lag in heat diffusion, as described by non-Fourier theory, which delays the thermal response and dampens the instantaneous heat flux at the boundary, leading to a lower effective temperature gradient and a consequent decline in $\left\{ {Nu_{x} \left( {{\mathrm{Re}}_{x} } \right)^{ - 1/2} } \right\}$. Table 7 explains the impression of various factors on motility number $\left\{ {Nn_{x} \left( {{\mathrm{Re}}_{x} } \right)^{ - 1/2} } \right\}$. With growth in (*Lb*) and Peclet number there is reduction in $\left\{ {Nn_{x} \left( {{\mathrm{Re}}_{x} } \right)^{ - 1/2} } \right\}$. The lessening in $\left\{ {Nn_{x} \left( {{\mathrm{Re}}_{x} } \right)^{ - 1/2} } \right\}$ with an intensification in both (*Lb*) and (*Pe*) is a consequence of diminished microbial transport efficiency relative to the dominant macroscopic flow mechanisms. A rise in (*Lb*) signifies a lower effective diffusivity of motile microorganisms compared to momentum diffusivity, meaning microbial swimming is less effective at dispersing cells through the fluid. This inherently restricts the ability of microorganisms to actively spread, thereby lowering the measure of their self-propelled motility. Simultaneously, an elevated (*Pe*) indicates that advective transport by the bulk fluid flow overwhelms the diffusive and self-driven motion of the microbes. In this regime, microorganisms are primarily swept along by the mainstream convection, which suppresses their autonomous swimming contribution to overall transport. The combined effect is that both the inherent dispersal capacity (via Lewis number) and the relative influence of self-propulsion (via Peclet number) are curtailed, leading to a net reduction in $\left\{ {Nn_{x} \left( {{\mathrm{Re}}_{x} } \right)^{ - 1/2} } \right\}$ as the system becomes increasingly dominated by external fluid forces rather than active microbial movement.

**Table 4:** Variation in $C_{x}$ via $\alpha$, $M$ and $K$

$\alpha$	$M$	$K$	$C_{x}$
0.1			1.497654
0.2			1.518647
0.3			1.530876
	0.1		1.968636
	0.2		1.975384
	0.3		1.985487
		0.1	1.565487
		0.2	1.587663
		0.3	1.603254

**Table 5:** Variation in $C_{y}$ via $\alpha$, $M$ and $K$

$\alpha$	$M$	$K$	$C_{y}$
0.1			1.879875
0.3			1.889875
0.5			1.896557
	0.1		1.846548
	0.3		1.865321
	0.5		1.886514
		0.1	1.913654
		0.3	1.932558
		0.5	1.956987

**Table 6:** Variation in $Nu$ via $Bi$, $Nt$, $Nb$ and $\delta_{T}$

$Bi$	$Nt$	$Nb$	$\delta_{T}$	$Nu$
0.1				1.565412
0.2				1.589875
0.3				1.603289
	0.1			1.876423
	0.2			1.854697
	0.3			1.836598
		0.1		1.565984
		0.2		1.603298
		0.3		1.646987
			0.1	1.498726
			0.2	1.486531
			0.3	1.479816

**Table 7:** Variation in *Nn* via *Lb* and *Pe*

*Lb*	*Pe*	*Nn*
0.1		1.875461
0.2		1.856987
0.3		1.836984
	0.1	1.598756
	0.2	1.556984
	0.3	1.513698

## Conclusions

This work numerically explores the steady MHD flow of nanofluid on the porous medium. The model integrates the combined effects of thermophoresis and Brownian motion to accurately describe nanoparticle behavior. It further analyzes the influence of Arrhenius activation energy on chemically reactive species. The thermal layer at borderline is governed by a convective heating condition, while the concentration field is subjected to a physically realistic zero mass flux condition at the boundary. After comprehensive analysis of work it has revealed that:-

- With growth in magnetic factor, inter-particle spacing and porosity factor there is drop in primary and secondary velocities.
- Both the primary and secondary velocities augmented with growth in radius of nanoparticles.
- Thermal profiles augmented with escalation in Brownian motion factor, thermal Biot number, thermophoresis factor and magnetic factor while declined with augmentation in thermal relaxation time factor.
- Concentration panels augmented with growth in thermophoresis factor and activation energy factor while decayed with rise in Schmidt number, chemical reactivity parameter and Brownian motion factor.
- A comparative analysis with established results confirms the validity and accuracy of the present model. The close agreement between our numerical outputs and the published data verifies the correctness of the solution methodology and the physical consistency of the formulated problem.

### Limitation and future suggestions

This work offers a validated multi-physics framework and design insights for nanofluid systems; temperature-dependent properties and the dynamics of nanoparticle aggregation should be incorporated into future research, and experimental validation is still necessary to turn these theoretical findings into useful applications.

## Data Availability

The data that support the findings of this study are available from the corresponding author upon reasonable request.

## List of symbols

*u*, *v*, *w*: Flow components [m s^−1^]
*x*, *y*, *z*: Coordinate axes [m]
$$\sigma$$: Electrical conductivity [S m^−1^]
$\mu$: Dynamic viscosity [kg m^−1^ s^−1^]
$$K^{*}$$: Porosity coefficient [m^2^]
$B_{0}$: Magnetic field intensity $\left[ {{\mathrm{kg\,s}}^{ - 2} \,{\mathrm{A}}^{ - 1} } \right]$
$$C_{p}$$: Specific heat $$\left[ {{\mathrm{J\,kg}}^{ - 1} \,{\mathrm{K}}^{ - 1} } \right]$$
$$D_{T}$$: Thermophoretic coefficient $$\left[ {{\mathrm{m}}^{2} \,{\mathrm{s}}^{ - 1} \,{\mathrm{K}}^{ - 1} } \right]$$
$\lambda_{T}$: Dimensional thermal relaxation time factor [s]
$\lambda_{C}$: Dimensional mass relaxation time factor [s]
$K_{1}$: Chemical reaction coefficient [s^−1^]
$E_{a}$: Activation energy coefficient $\left[ {{\mathrm{J\,mol}}^{ - 1} } \right]$
*n*: Power index [–]
$W_{c}$: Maximum cell swimming speed $\left[ {{\mathrm{m\,s}}^{ - 1} } \right]$
$$D_{m}$$: Microorganism diffusion $$\left[ {{\mathrm{m}}^{2} \,{\mathrm{s}}^{ - 1} } \right]$$
$h_{f}$: Heat transfer coefficient $\left[ {{\mathrm{W\,m}}^{ - 2} \,{\mathrm{K}}^{ - 1} } \right]$
$$d_{p}$$: Nanoparticle radius [m]
*h*: Inter-particle spacing [m]
$\varphi$: Nanoparticle volume fraction [–]
$$K$$: Porosity factor [–]
$$M$$: Magnetic parameter [–]
$$\alpha$$: Ratio factor [–]
*Bi*: Thermal Biot number [–]
Pr: Prandtl number [–]
$\delta_{C}$: Mass relaxation time factor [–]
$\delta_{T}$: Thermal relaxation time factor [–]
*Pe*: Peclet number [–]
*K*_*r*_: Chemical reaction [–]
*Sc*: Schmidt number [–]
*Nb*: Brownian motion parameter [–]
*E*: Activation energy factor [–]
*Lb*: Lewis number [–]
*Nt*: Thermophoresis parameter [–]
